# Cross Mean Annual Runoff Pseudo-Elasticity of Entropy for Quaternary Catchments of the Upper Vaal Catchment in South Africa

**DOI:** 10.3390/e20040281

**Published:** 2018-04-13

**Authors:** Masengo Ilunga

**Affiliations:** Department of Civil and Chemical Engineering, College of Science, Engineering and Technology, Florida Campus, University of South Africa, Florida 1710, South Africa; ilungm@unisa.ac.za; Tel.: +27-11-471-2791

**Keywords:** entropy, cross elasticity, mean annual runoff, water resources, resilience, quaternary catchment, complement, substitute

## Abstract

This study focuses preliminarily on the intra-tertiary catchment (TC) assessment of cross MAR pseudo-elasticity of entropy, which determines the impact of changes in MAR for a quaternary catchment (QC) on the entropy of another (other) QC(s). The TCs of the Upper Vaal catchment were used preliminarily for this assessment and surface water resources (WR) of South Africa of 1990 (WR90), of 2005 (WR2005) and of 2012 (WR2012) data sets were used. The TCs are grouped into three secondary catchments, i.e., downstream of Vaal Dam, upstrream of Vaal dam and Wilge. It is revealed that, there are linkages in terms of mean annual runoff (MAR) between QCs; which could be complements (negative cross elasticity) or substitutes (positive cross elasticity). It is shown that cross MAR pseudo-elasticity can be translated into correlation strength between QC pairs; i.e., high cross elasticity (low catchment resilience) and low cross elasticity (high catchment resilience). Implicitly, catchment resilience is shown to be associated with the risk of vulnerability (or sustainability level) of water resources, in terms of MAR, which is generally low (or high). Besides, for each TC, the dominance (of complements or substitutes) and the global highest cross MAR elasticity are determined. The overall average cross MAR elasticity of QCs for each TC was shown to be in the zone of tolerable entropy, hence the zone of functioning resilience. This could assure that water resources remained fairly sustainable in TCs that form the secondary catchments of the Upper Vaal. Cross MAR pseudo-elasticity concept could be further extended to an intra-secondary catchment assessment.

## 1. Introduction

Elasticity, a concept borrowed from economic sciences has been used in hydrology and water resources to determine a relative change in a variable, e.g., rainfall, with respect to the change in runoff generated [1,2,3]. When rainfall is considered to be the most influential variable for generating runoff (e.g., bivariate rainfall-runoff relationships), rainfall elasticity of streamflow is generally positive, i.e., an increase in rainfall is translated into an increase in runoff [1]. This is contrary to the pure economic law on the price elasticity of demand, which is always negative [4,5], however market forces may dictate the sign (positive or negative) of price elasticity [5,6]. Nonetheless other parameters have been shown to influence positively or negatively rainfall elasticity of streamflow, e.g., temperature/evapotranspiration [7,8,9], land use, water use [10]. The notion of elasticity has been linked recently to entropy concept to assess catchment resilience, i.e., mean annual runoff (MAR) pseudo elasticity [11]. The notion of pseudo-elasticity of entropy was derived for linear regression models and measured the relative change in entropy with respect to the relative change in MAR for tertiary catchments (TC), which are comprised of quaternary catchments (QCs). For the specific case of TCs of the Upper Vaal, MAR pseudo-elasticity of entropy was shown to be relatively positive when considering regression models. In hydrology and water resources, the computation of elasticity is usually related to the same catchment that undergoes change/transformation, i.e., what will be the relative change in runoff/streamflow corresponding to 1% change in rainfall for a given catchment? Likewise, for MAR pseudo-elasticity of entropy, a certain % change in entropy will be associated with 1% change in MAR, for a given catchment. Pseudo-elasticity and elasticity concepts in water related studies could be referred to as self/own-elasticities since changes in the different variables have been formulated with respect to the same catchment. Self/own-elasticity is commonly used to mean elasticity [12,13,14]. Moreover, in hydrology and water resources, elasticity/pseudo-elasticity concept for a given catchment is often defined without the influence of changes in variables from other catchments. The current paper takes this opportunity by assessing how changes in MAR for a given catchment will likely impact on changes in entropy associated with MAR for another (other) catchment(s). Hence this was done by introducing explicitly the notion of cross elasticity of entropy associated with hydrological change (i.e., change in MAR) occurring in two different catchments belonging to the same hydrological region. In this study, the cross MAR pseudo-elasticity of entropy is defined as the ratio between the change in entropy of a given QC and the change in MAR of another catchment. Cross elasticity is assessed preliminarily at the smaller scale of catchment, i.e., QC. Currently, there is almost no literature in hydrology and water resources that deals explicitly with cross elasticity concept. Cross elasticity concept originated from economy, i.e., cross price-elasticity of demand and shows how the change in price of a given good is likely linked to the demand of another good [12,13,14,15,16]; as opposed to (self/own)-elasticity, which considers the change in price and demand for the same good. Cross price-elasticity of demand yields complementary goods when price and demand vary in different directions and to substitutable goods when the signs are the same [13,15]. Complementary goods and substitutable goods are usually referred to as complements and substitutes respectively [15,17]. Cross elasticity concept was later applied to other fields; e.g., transportation [6,18], marketing [13], electricity [19], etc. The following could also support the introduction of cross elasticity concept in hydrology and water resources:

- In the same hydrological zone (TC) and beyond, different QCs are not considered in isolation (hence are interdependent) and are usually subject to activities such as domestic, industrial, social and economic [20], which are likely to impact on MAR [11]. For instance exaggerated inefficient water use from one part of the catchment may impact over time on other parts of the catchment, e.g., uneven distribution of water availability coupled with unbalanced water demand/supply; change in MAR, etc.
- Water strategy exists to enable proper management and efficient use of water resources in a given region [21]. Thus an integrated water management resource approach is always required to balance water demand and supply. This is possible only if water is well managed at the smaller scale; i.e., quaternary level, within the TC and that water management takes into consideration of how changes in MAR for one QC is likely to affect the changes in another (other) catchment(s). Water resources could be explored from specific catchments of the region without compromising the sustainability of water resources in the region.
- In reality, water transfers can be made intermittently among different catchments, in view of balancing water demand and supply. In a complementary way, water resources could be jointly sustainably used among two or more catchments. These could be seen as “complementary” catchments. Alternatively, water resources from specific catchments could be used to support several activities (domestic, industrial, social and economic) in other catchments. Catchments supplying water could be seen as “substitutes” of those receiving water. A practical example is the Vaal catchment of South Africa (with the Vaal dam) that supplies water from its catchments to several other catchments in Gauteng and beyond. Hence it is a fact that the Upper Vaal catchment impacts on the water balance of several catchments within South Africa and beyond [20].

Inspired by an economic aspect, e.g., [5], this study suggested that when water resources from two catchments can be used jointly sustainably to support their activities, the catchments will be referred to as complementary catchments (complements). One catchment could enhance the insufficient yield of another catchment, but not the opposite; hence the former will be seen as “complement” of the latter. When water resources from one catchment are used preferably over another (other) catchment(s) and vice-versa, these catchments will be referred to as substitutable catchments (substitutes). Water resources from one catchment could used to support another catchment, but not the opposite; the former will be referred to as “substitute” of the latter.

In the light of the above, this study investigates preliminarily, the change in uncertainty of MAR for a given QC with respect to the change in MAR of another (other) QC(s), hence cross MAR elasticity of entropy. Climatic conditions and human activities may cause changes in hydrological variables such as streamflow (runoff) [20,22,23,24,25,26], which has been associated with a degree of uncertainty expressed as Shannon entropy, e.g., [23,25,27,28]. As mentioned earlier, natural climatic conditions [7,8,9,11,29,30,31] and/or human activities [10] may influence positively or negatively streamflow elasticity. Nonetheless, naturalized streamflows, thus naturalized MAR plays an important role in South Africa for water resources planning, development and management; hydraulic structure control, natural variability associated with climatic conditions; and ecological water requirements [32]. Hence surface water resources of South Africa of 1990 (WR90), of 2005 (WR2005), and of 2012 (WR2012) usually present the naturalized MAR data of the different catchments, that include the Upper Vaal catchment. In their derivation, naturalized flows are observed flows adjusted with the net effect of upstream land use changes [33]. It is also acknowledged that a problem of unknown origin may occur during streamflow adjustments at a given gauge [33]. Meteorological data with limited human influence could be focused on climatic controls [31]. MAR is assumed to be relevant to a catchment under virgin conditions, i.e., prior to any human activities that have influenced the hydrology of the catchment [33]. Therefore naturalized MAR could assume adjustments of observed flows with quantifiably manageable land use changes. For that, cross MAR pseudo-elasticity could be assumed dependent on natural factors, e.g., climatic variations (temperature, rainfall, evapotranspiration), natural vegetation, natural wetlands and aquifers, soil type, topography, etc. Data at QC level is very important since a QC is usually considered as the smallest unit for hydrological studies, at tertiary, secondary and primary level. The Upper Vaal catchment is made of three secondary catchments, which comprise three TCs each. TCs are finally subdivided into QCs.

The implications of cross elasticity (in comparison with self-elasticity) on catchment resilience were assessed in so far as water resources are concerned. Cross elasticity of entropy was applied to the Upper Vaal catchment and the spatial assessment of cross MAR pseudo-elasticity at QC level, which is fundamental for tertiary and secondary catchments, was also investigated. It is noted that the assessment of spatial *distribution* (*variation*) of rainfall elasticity of streamflow [2,3,7,8,9,29,30,31], and entropy associated with streamflow [22,23] and with precipitation [34,35,36,37] have been documented. The use of maps for the spatial distribution analysis of the catchment under investigation, is common. This study is built on MAR pseudo-elasticity concept that was introduced recently [11]. It is a preliminary study that investigates cross MAR pseudo-elasticity concept within TCs. It is natural to consider QCs, which constitute a TC. It does not deal yet in detail with cross MAR pseudo-elasticity among TCs, within secondary catchments. Hence a separate study should be conducted at secondary level to determine the impact of change in entropy of a TC with respect to the change in MAR at another TC. Moreover, the individual as well as the combined effect of natural factors on cross MAR pseudo elasticity could be further investigated.

The paper is organized as follows: the first section gives an overview on cross elasticity, which focuses on cross price-elasticity concept from economics, as compared with elasticity (self-/own-elasticity). Main characteristics of the concept are given and its applications to other fields are outlined. The second section explores the main characteristics of cross-elasticity and extends the concept to hydrology and water resources, by focusing preliminarily on the change in MAR and its uncertainty (entropy), hence cross MAR pseudo-elasticity of entropy. MAR is a very important hydrological variable. The third section gives the methods for assessing cross MAR elasticity of entropy and its implication for catchment resilience/sustainability of water resources. The fourth section applies the methods to the Upper Vaal catchment; hereby presents the results and discussion on cross MAR pseudo-elasticity, by outlining the implications on hydrological resilience. The last section summarizes the findings from the previous section and presents recommendations and outlines further research.

In what follows, the prefix pseudo will be sometimes omitted from elasticity. Self/own-elasticity will mean elasticity as compared to cross elasticity. Hence cross MAR pseudo-elasticity will often mean cross MAR elasticity. MAR will mean naturalized mean annual runoff. Complementary catchments and substitutable catchments will be referred to as complements and substitutes respectively. Uncertainty and entropy could mean the same. Upper Vaal, Upper Vaal catchment and Upper Vaal region will be used interchangeably.

## 2. Overview on Cross Elasticity Concept

From an economic point of view, price-elasticity has been defined as the change in the demand of a good (brand) over the change in price of the same good [14,15]. Elasticity is referred some times to as self- or own-elasticity [13,14], since these changes are assessed with respect to the same good (brand). When changes are assessed with regard to two different goods, the term cross price-elasticity is used; i.e., how the demand of a good is linked to the change in price of another good [5]. The importance of cross price-elasticity has been shown to give power to the consumer for switching to other brand(s), when one good becomes unaffordable or rare, for instance [13,15].

Some of the important characteristics of cross price-elasticity are as follows: firstly a positive cross elasticity between two brands shows that these brands are substitutable; secondly brands are complementary when their cross price-elasticities vary in opposite direction (i.e., negative) [5,14]; and lastly the two brands do not compete at all when their cross elasticities are null [15]. These characteristics among brands are referred to as substitutability, complementarity and independence respectively [15]. The context of the market determines the extent to which substitutable, complementary goods could both appear together in the perpetual space [15]. It was shown also that a good (palm oil) could be a substitute for another good (soy oil) and not the opposite [5]. In specific situations, complementarity can be related to the purchase of two goods on a single buying opportunity, while substitutability can be related to switching between goods of interpurchase times [15]. The higher the cross elasticity between two brands, the more correlated the goods should be [13,14,15]. Results on cross-elasticity are usually summarized into a matrix called self and cross elasticity matrix; which was shown to be asymmetric generally with the upper left diagonal of the matrix being the line of asymmetry, for which the diagonal entries are self-elasticities [14,38]. The strength of a brand with respect to the interbrand price competition is measured by the amount of asymmetry [17]. In exceptional cases, constraints could be imposed to have a symmetric cross elasticity matrix [17,39].

These characteristics are so appealing that they were adapted to the case of water resources sustainability; in particular catchment resilience assessed using entropy associated with MAR. For instance, water managers could decide to switch to a water surplus catchment to ensure good water demand/supply balance in the region (substitutability). Hence water transfers among different catchments could be possible for water sustainability of the hydrological region. Water managers could also decide to use jointly water resources from different catchments (complementarity). Water transferred from one catchment to another catchment and possibly vice-versa, may not be necessarily the same (asymmetry); hence the impact of change in MAR of a catchment on entropy of another catchment and vice-versa may not necessarily be the same. MAR is one of the important hydrological variables for catchment resilience or water resources sustainability since the yield of a catchment depends on MAR [11]. Hence for the purpose of the current study, cross MAR pseudo-elasticity was used to assess how changes in uncertainty for a given catchment were possibly linked to changes in MAR for another catchment, within the same TC. In line with Ilunga (2017), the link between cross elasticity concept and catchment resilience in terms of water resources sustainability, was determined by using the concept of linear zoning of catchment resilience. Similar to selected studies on entropy of streamflow [22,23], entropy of rainfall [35,36,37] and in particular rainfall elasticity of streamflow [2,3,7,8,9,29,30,31], spatial distribution (variation) assessment of cross MAR pseudo-elasticity across the Upper Vaal catchment was carried out by using a map representation. This is a preliminarily study that introduces cross MAR pseudo-elasticity of entropy at QCs level within a TC and could be further extended to TCs within secondary catchments.

## 3. Cross MAR Pseudo-Elasticity in Hydrology and Water Resources

The literature of cross elasticity concept is almost inexistent in hydrology and water resources. As outlined earlier, MAR pseudo-elasticity of entropy concept has been introduced recently [11] and on which cross-MAR elasticity is built. A bivariate model (i.e., linear model) was used to derive MAR pseudo-elasticity of entropy for the different TCs of the Upper Vaal catchment. In particular, MAR pseudo-elasticity was interpreted as the regression coefficient of the linear regression. MAR pseudo-elasticity of entropy (*ε_i_*) was defined as the ratio between relative changes in uncertainty associated with MAR and changes in MAR for different QCs of a given TC.

It is useful to give important equations that are critical in the determination of cross MAR elasticity as adapted from Ilunga [11].

Equation (1) below is derived from the Shannon entropy index of the variable *z_i_*, and shows the uncertainty associated with *z_i_*. Entropy can be written as *H*(*z_i_*). The variable z*_i_* can be MAR for a specific *i*th QC in a given TC. The *i* values (*i* = 1, 2, 3, …, *k*) represent the *i* QCs in the TC of the Upper Vaal catchment . Each QC contributes to the total MAR of their TC.

(1) $$H(z_{i})=-\frac{z_{i}}{Z}\log\frac{z_{i}}{Z}$$

In Equation (1), the base of the logarithm is the unit of *H*(*z_i_*). It is in bits if the base is 2, in Napiers if the base is *e*, and in decibels (dB) if the base is 10. If *Z* is MAR of the TC in either WR90, 2005 or WR2012 data set, therefore $Z=\sum_{i=1}^{k}z_{i}$.

For a specific *i*th QC in a given TC, the relative change/variation in MAR and relative change in entropy are given by Equations (2) and (3) respectively as shown below when using W90 and W2005 data sets:

(2) $$\delta_{i}=\delta (z_{i})=[\frac{z_{i}{}_{\left(WR2005\right)}-z_{i(WR90)}}{z_{i(WR90)}}]\times 100$$

(3) $$\delta H_{i}=\delta H(z_{i})=[\frac{H(z_{iWR2005})-H(z_{iWR90})}{H(z_{iWR90})}]\times 100$$

where *δ*(*z_i_*), *δH*(*z_i_*) are the relative change/variation in MAR and relative change/variation in entropy for a specific *i-*th QC respectively. The variable *z_i_* is MAR as defined earlier. 

Similar Equations to (2) and (3) can be established for WR2005/WR2012.

For hydrological changes occurring between 2 data sets, the MAR pseudo-elasticity for the *i*-th QC of a given TC, is given by Equation (4):

(4) $$\varepsilon_{i}=\frac{\delta H_{i}}{\delta_{i}}$$

where *δH_i_* is the relative change in entropy associated with MAR; *δ_i_* is the relative change in MAR for the *i*-th QC in the TC.

Equation (4) can be referred to as self/own-MAR pseudo elasticity of entropy for the *i*-th QC since changes in the equation are defined with reference to the same *i*-th QC. Changes are expressed in relative terms. Hence cross MAR pseudo-elasticity (*ε_ij_*) can be defined as the ratio between the change in entropy of *i*-th QC and the change in MAR of *j*-th QC. Hence cross MAR pseudo-elasticity (*ε_ij_*) is given by Equation (5):

(5) $$\varepsilon_{ij}=\frac{\delta H_{i}}{\delta_{j}}$$

Equation (5) determines the % change in entropy of *i*-th QC associated with a 1% change in MAR of the *j*-th QC.

Conversely, cross MAR pseudo-elasticity (*ε_ji_*) is the ratio between the change in entropy of *j*-th QC and the change in MAR of *i*-th QC. This is translated into Equation (6) as given below:

(6) $$\varepsilon_{ji}=\frac{\delta H_{j}}{\delta_{i}}$$

The terms in Equation (6) can be defined in a similar way as in Equation (4).

In hydrology and water resources studies, rainfall elasticity of streamflow [1] and MAR pseudo elasticity of entropy for bivariate relationships [11] were generally positive. This is not in line with the ideal economic law of the price-elasticity of demand, which is negative [4,5]. However, other hydro-climatic variables (i.e., temperature and evapotranspiration) [9] and human activities (i.e., land use, water use, etc.) [10] could impact positively or negatively rainfall elasticity. Pseudo-elasticity of entropy varied in opposite direction with mean annual evaporation, and in the same direction with mean annual precipitation for TCs of the Upper Vaal [11]. A positive MAR pseudo-elasticity of entropy means that an increase/decrease in entropy is associated with an increase/decrease in MAR. A negative MAR pseudo-elasticity of entropy implies the variations in entropy and in MAR are of opposite signs. Similar to economic concept, two catchments will be said “complements” in terms of water resource sustainability, when the cross-MAR elasticities are negative. The sustainability of water resources is characterized by the level of catchment resilience defined with respect to the uncertainty associated with MAR [11]. Complementarity refers to the increase/decrease in entropy for one catchment, linked to the decrease/increase in MAR for another catchment and vice-versa. Hence water resources could be used jointly from the 2 catchments to balance water demand/supply. When cross MAR elasticities are positive, QCs could be regarded as substitutes, in terms of water resource sustainability (catchment resilience). In this case, water transfer between different QCs could also be promoted to balance water demand/supply. Substitutability is referred to the increase/decrease in entropy for one catchment linked to the increase/decrease in MAR for the other catchment and vice-versa. There could be a situation where one catchment could be a substitute/complement for another catchment, but not vice-versa [5]. Complementarity and substitutability for different QCs should always be comprehended within the zone of acceptable uncertainty (entropy), i.e., the zone of functioning resilience associated with MAR. The zone of acceptable uncertainty that defines catchment resilience was determined by the interval (10%, 25%) increase/decrease for pseudo elasticity; when considering a change in 10% MAR [11]. That is, MAR pseudo-elasticity of entropy should vary between −25% and 25%. This interval was confirmed valid when a statistical analysis was conducted on the significance of MAR pseudo-elasticity, within the context of linear regression. Hence, the cross MAR elasticity of entropy associated with MAR could be assumed acceptable, if it could fall into the above-mentioned interval.

The following could also enhance the introduction of cross MAR elasticity concept:

At a lower scale, the different QCs in a TC are not considered in isolation or may have a certain level of interdependence, as far as water resources are concerned. Streamflows from one catchment may have an impact in one way or the other on the runoff of another catchment. This interdependence may cause a change in runoff in the different catchments and change in uncertainty associated with MAR. For example surrounding catchments of the Vaal dam catchments may contribute to the inflow into Vaal dam. In return, water stored in the Vaal dam will be supplied and contribute to several activities in the QCs of the TCs and beyond. 

At a relatively larger scale, water transfers can be made among different water management areas, in view of balancing water demand and supply between two or several catchments, i.e., in the Upper Vaal, water resources should be well managed and co-ordinated with other interdependent catchments, in a way to have an integrated water resource system [20]. Hence, there is a need for water resources to be properly managed strategically at the smaller scale; i.e., quaternary level, within the TC as well as at a larger scale such as secondary and primary catchments. Water management should take into consideration of how the change in MAR of a given catchment is likely to affect the change in uncertainty associated with MAR in another catchment, within the same hydrological zone and beyond.

## 4. Methods and Data Availability

### 4.1. Data Used

**Figure 1:**
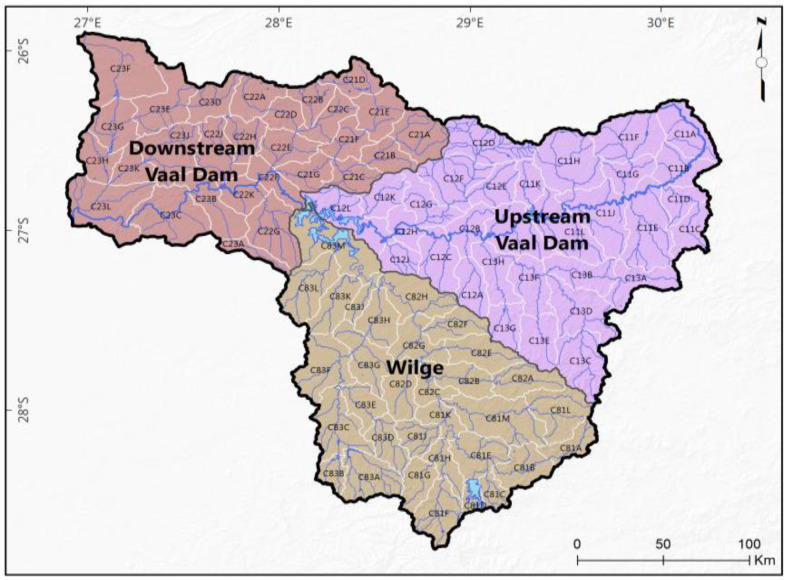
Quaternary, tertiary and secondary catchments of the Upper Vaal catchment [11].

The Upper Vaal catchment is one of the three parts of the Vaal catchment (i.e., a primary catchment) of South Africa. The two other parts of the Vaal catchment are the Lower Vaal and Middle Vaal. The Upper Vaal falls partly into the Gauteng province, which is considered as the economic hub of the country. The Upper Vaal catchment is situated approximately between 26.5° and 30.5° E longitudes and between 25.2° and 26.2° S latitudes (Figure 1). 

The Upper Vaal is subdivided into three secondary catchments: upstream of Vaal dam (C1), downstream of Vaal dam (C2) and Wilge (C8). Upstream of Vaal dam comprises the following TCs: C11, C12 and C13. Downstream of Vaal dam is formed of the following TCs: C21, C22 and C23. Wilge is subdivided into the following TCs: C81, C82 and C83. Each TC is subdivided into several QCs. A QC is considered to be the basic areal unit for WR90, WR2005 and WR2012 data sets of South Africa as published by Water Research Commission [40]. According to the nomenclature used by the Water Research Commission of South Africa (WRC), a given QC, e.g., C11A means that C is the primary catchment (i.e., Vaal catchment), 1 is the secondary catchment, 1 refers to the TC and the letter at the end is specific to the QC. Upper case letters are usually used in this nomenclature. A good summary of data used for the Upper Vaal was given previously [11]. Nonetheless, of importance is the number of QCs for each TC (Table 1). Details pertaining to catchment areas of QCs, MAR, mean annual precipitation (MAP), mean annual evaporation (MAE), are detailed in surface water resources of 2012 (WR2012) reports, which are the latest publications for water resources of South Africa [40]. MAP and MAE are around 700 mm and 1400 mm. The climate in the Upper Vaal is temperate and fairly uniform, with strongly seasonal rainfall, occurring during the summer period. Savannah grassland, with sparse bushveld is the type of vegetation; the foothills of the Maluti constitute the main topographic feature; the soils are arable and dolomitic. The surface water resources of 2012 (WR2012) reports contained all three data sets; i.e., surface water resources of 1990 (WR90), of 2005 (WR2005) and of 2012 (WR2012). WR90 included MAR data from 1920 to 1989; WR2005 included MAR data from 1920 to 2004 and WR2012 contained MAR data from 1920 to 2012 [40]. The reader should use this reference for further details. The methods laid down in the next section will be performed for both hydrological changes between data sets WR90 and WR2005, and between WR2005 and WR2012. These changes were also written as WR90/WR2005 and WR2005/2012 respectively. MAR is assumed for a catchment under virgin conditions, i.e., without any human influence that have affected catchment hydrology [33]. 

In the above table, QC is quaternary catchment, MAR is mean annual runoff, WR90 is surface water resources of 1990 data sets, WR2005 is surface water resources of 2005 data sets, WR2012 is surface water resources of 2012 data sets.

**Figure 2:**
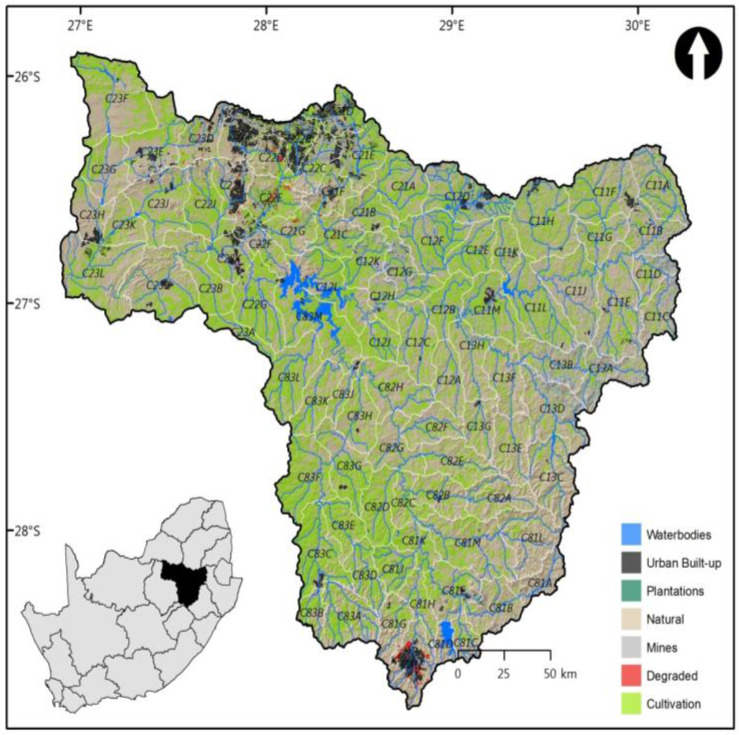
Land use/cover in the Upper Vaal of 2009.

Figure 2 shows the land use/cover of Upper Vaal catchment, with a total area of 55,565 km^2^. It is comprised of natural, cultivation, degraded, urban built-up, water bodies and plantations. The natural part is the most dominant part and constitutes more than the half of the total area. It is followed by the water bodies and the urban built-up area which constitute, each, approximately 3% of the total area. The urban built-up and mining areas (coal and gold) are mostly confined into the downstream of Vaal, particularly, in the northern and the north-west parts of the Upper Vaal catchment. Due to the presence of the large numbers of financial and business service institutions and head offices in Gauteng, the financial sector (with a number of business institutions in Gauteng) plays an important role in the Upper Vaal catchment. 

In the next section, for the reason given earlier, the method is mainly based on cross MAR pseudo-elasticity for QCs, within TCs. 

### 4.2. Methods

#### 4.2.1. Complementarity and Substitutability of QCs

For different QCs in a given TC, MAR pseudo-elasticities and cross MAR pseudo-elasticities were computed according to Equations (1)–(6). The matrix of cross MAR elasticities could be set up and was assessed for symmetry/asymmetry. The matrix is a square matrix *k* × *k*, where *k* is the number of QCs in a given TC. For a specific QC pair, matrix symmetry helped to assess whether or not the change in MAR of one QC had the same impact on the change in entropy of the other QC, and vice-versa. This yielded the determination of complementarity and substitutability of QC pairs. The level of complementarity and substitutability of QC pairs was determined from the signs and magnitudes of cross MAR elasticities of entropy, as explained previously. Hence in this manner, complementary QCs (complements) and substitutable QCs (substitutes) could be determined. In line with Ilunga (2017), three zones of catchment resilience were essentially determined for cross MAR pseudo-elasticity, based on the linear resilience approach zoning:

- Zone of high uncertainty: this is a zone of high entropy and assumes that water resources are not sustainable. It corresponds to higher values of cross elasticities, which are the result of relatively small changes in MAR associated with relatively higher values of entropy. This zone was considered for cross elasticity values above 25%, i.e., 2.5 in unit terms and was characterized with low resilience. Substitutability of QCs becomes impractical due to the higher magnitudes of relative changes in entropy as compared to the changes in MAR and these changes are of the same sign. Natural factors such as climatic conditions, topography, soil type, natural vegetation, etc. could dominate.
- Zone of functioning resilience: this is a zone of tolerable uncertainty and assumes that water resources could be managed sustainably. It corresponds to the values of cross elasticities, which are derived from small changes in MAR associated with reasonable changes in entropy. This zone was considered for cross elasticity values between −25% and 25%, i.e., −2.5 and −2.5 units. Substitutability and complementarity of QCs for water resources planning and management could be sustainable.
- Zone of uniformity: this is a zone, where a catchment is prompt to water resources vulnerability due to relatively low values of entropy and water resources cannot be sustainable if further changes in MAR occur. The zone corresponded to values of cross elasticity below −25%, i.e.,−2.5 in unit terms. Complementarity of QCs could be assumed impractical due to higher relative changes in entropy as compared to the change in MAR and these changes could be of opposite directions. Once again natural conditions could also prevail over land use/cover changes. The first and the last zones are characterized by low resilience.

In general, the interval (−25%, 25%) was used for both elasticities [11] and cross elasticities to assess catchment resilience/water resources sustainability.

#### 4.2.2. Cross MAR Pseudo-Elasticities under Constant Change in MAR/Entropy

For any *j*-th column of the matrix, cross MAR pseudo-elasticity values were computed from the changes in entropy of the k QCs and a change in MAR of the *j*-th QC. Each value shows the extent to which a constant change in MAR for the *j*-th QC affects the entropy of remaining QCs. The average of these values is given by Equation (7) below:

(7) $$\varepsilon_{j}=(1/k)\sum_{i=1}^{k}\varepsilon_{ij}\;(j=1,2,\ldots ,k)$$

Likewise, for any *i*-th row of the matrix, the average cross MAR elasticities computed from a change in entropy of the *i*-th QC and the changes in MAR of the *k* QCs, is given by Equation (8) below:

(8) $$\varepsilon_{i}=(1/k)\sum_{j=1}^{k}\varepsilon_{ij}\;(i=1,2,\ldots ,k)$$

*ε_j_* shows on average how a constant change in MAR of the *j*-th QC may impact on entropy of *k* QCs, whereas *ε_i_* shows on average how changes in MAR of *k* QCs may impact on the *i*-th QC, such that the change in its entropy remains constant. If *ε_i_* (*ε_j_*) is positive, then the positive cross elasticities dominate over the negative cross-elasticities. Othewise *ε_i_* (*ε_j_*) is negative, hence the negative cross elasticities dominate over the positive cross elasticities. Equations (7) and (8) were useful in drawing the map of the spatial distribution of cross MAR elasticities of the different QCs, as explained in Section 4.2.6.

The average cross MAR pseudo-elasticity (*ε_p_*) for any *p*-th TC in a secondary catchment as presented in Equation (9) below, was computed from either Equations (7) or (8):

(9) $$\varepsilon_{p}=\frac{\sum \varepsilon_{i}}{k}\;(p=1,\ldots ,3)$$

where *k* is the number of QCs in a given TC.

Similarly, the overall average of cross MAR pseudo-elasticity for a given secondary catchment was roughly computed from Equation (9) by using an average of three TCs. As mentioned in the previous section, the number of TCs in each secondary catchment is 3. Normally, a detailed study should be conducted at secondary catchment level to determine the impact of the change in entropy of one TC with respect to the change in MAR at another TC.

#### 4.2.3. Strengths of Correlation between QCs

Similar to economics [15], cross MAR elasticity could show the correlation between QCs, in terms of water resources sustainability/vulnerability. The higher the cross MAR elasticity between two catchments, the more correlated the two catchments should be. That is, a strong correlation could correspond to low catchment resilience (high elastic instability of QCs) or zone of high uncertainty. Conversely, an acceptable catchment resilience could be associated with low cross MAR pseudo-elasticity. This could imply that resilient catchments could be correlated (linked) through the zone of acceptable uncertainty (elastic stability) for water resources sustainability. Therefore, in terms of MAR, high and weak cross elasticity of entropy will imply promptness to vulnerable and sustainable water resources respectively. Using the linear zoning of resilience for the different QCs as described earlier, the following conditions for the correlation strength between *i*-th and *j*-th QCs were applied:

- Strong correlation between QCs *i* and *j* (low catchment resilience or vulnerability): *ε_ij_*, *ε_ji_* < −2.5 or *ε_ij_*, *ε_ji_* > 2.5.
- Low correlation between QCs *i* and *j* (high catchment resilience or sustainability): *ε_ij_*, *ε_ji_* ≥ −2.5 and *ε_ij_*, *ε_ji_* ≤ 2.5. (This includes the case of complete independence between QCs, i.e., where *ε_ij_*, *ε_ji_* = 0).
- In % form, the cross elasticity thresholds −2.5 and 2.5 are −25% and 25%, respectively.

#### 4.2.4. Dominance between Complements and Substitutes

The number of occurrences of complements and substitutes in the different QCs pairs enabled to derive their frequencies, which were determined from the *k* × *k* entries of the matrix. Frequency in % showed the dominance between complements and substitutes, in each TC. This indicates whether or not water resources could be used jointly from different QCs or predominantly from specific catchments, alternatively to allow transfers among QCs.

#### 4.2.5. Catchment Vulnerability Risk 

As a flip side of resilience, catchment vulnerability could be seen as a risk for water resources not being sustainable, in terms of MAR. The focus is on strong cross MAR elasticities and their magnitudes within a TC, which may cause catchments having relatively low resilience. Catchment vulnerability was understood mainly in the context of the risk for a catchment not sustaining the yield, i.e., MAR. It shows the proportion of occurrences that a catchment could operate within the zone of elastic instability, i.e., either zone of high uncertainty or zone of uniformity [11]. In this way, the dominance of complementary catchments or substitutable catchments was determined.

Mathematically in terms of risk concept, catchment vulnerability (*r*), could be given by Equation (10):

(10) $$r=\frac{N}{k^{2}}$$

where *N* is the number of occurrences of strong cross MAR elasticities of entropy; *k* is the number of QCs in a TC.

Hence the level of assurance for a catchment not being vulnerable (*R*) will be defined by Equation (11)

(11) $$R=1-r=\frac{k^{2}-N}{k^{2}}$$

Lastly the highest cross elasticity per TC helped to assess the level of resilience. This is the highest variation in entropy versus change in MAR, which could be positive or negative. When highest cross MAR elasticities do not fall in the acceptable range of resilience, negative and positive values corresponded to the zone of uniformity (i.e., very low entropy) and zone of high entropy respectively. In other words, as explained previously cross MAR elasticity values were checked against the interval (−25%, 25%) for a change of 10% in MAR to decide whether the zone of functioning resilience/zone of acceptable entropy applied. Outside this range, water resources in QCs could be prompt to vulnerability unless found otherwise.

#### 4.2.6. Spatial Distribution of Cross MAR Pseudo-Elasiticities

Similar to precipitation elasticity of streamflow [2,3,7,8,9,29,30,31], the analysis of the spatial distribution (variation) of cross MAR pseudo-elasticities was carried out by using a map representation of the Upper Vaal catchment depicting the different QCs. Threshold values of elasticities (e.g., 1.0 to 2.0 in absolute values) derived from statistical analysis were used for spatial distribution analysis of streamflow elasticity [1] and at the most 1.0 to 2.5 [11]. For example, a value above 2.0 was adopted and used for elastic conditions of streamflow, hence below this value, streamflows were considered inelastic [2,29]. The intent of spatial distribution, in this study was simply to assess the differences in the cross MAR elasticities across Upper Vaal catchment. In the current study, the spatial analysis was confined mainly to the thresholds values (−2.5 and 2.5) for the zone of acceptable resilience, as defined earlier. The thresholds for the linear zoning approach of catchment resilience were verified statistically for the Upper Vaal by conducting a significance test [11] and were adapted to this study. Two cases were mainly considered for spatial distribution analysis: the average cross MAR pseudo-elasticity under constant change in MAR and cross MAR pseudo-elasticity under constant change in entropy at a given QC. Hence Equations (7) and (8) were used to determine the values of cross MAR elasticities that were drawn on the map of Upper Vaal catchment.

## 5. Results and Discussion

The assessment of cross MAR elasiticities, was based on the methods outlined in Section 4. In the tables presented later in the current section, the computations revealed that most QCs in the different TCs were dominated by positive MAR elasticity values. Complementarity and substitutability for the different QCs of the TC are summarized as shown, i.e., from Table 2, Table 3, Table 4, Table 5, Table 6, Table 7, Table 8, Table 9, Table 10, Table 11, Table 12, Table 13, Table 14, Table 15, Table 16, Table 17, Table 18 and Table 19. Hence resilient QCs (in terms of water resources) displayed generally weak cross elasticity of entropy values, as opposed to unstably elastic QCs. It was observed that some QC pairs displayed both positive and negative cross MAR elasticity of entropy. Hence in a QC pair, one catchment could be regarded as complement and the other as substitute, when dealing with their changes in MAR respectively. The analysis of cross MAR pseudo-elasticity and interpretation of results was focused on specific QC pairs and could be naturally extended to the remaining QC pairs. Nonetheless the spatial distribution gave further insights in the differences of cross MAR elasticity across Upper Vaal. Catchment vulnerability risk was also assessed to have implicitly the level of assurance for sustainable water resources. The analysis of cross MAR pseudo-elasticity in hydrology and water resources has been carried out preliminarily at QC level, as shown in this section. For each TC, the number of QCs determined the dimension of the cross MAR elasticity matrix and two matrices were presented in a tabular form (e.g., for C11, Table 2 and Table 3). The matrices are representative of hydrological changes between WR90 and WR2005, and between WR2005 and WR2012 data sets. The last row in the tables depicts the average cross elasticity at constant change in MAR of each QC and the last column corresponds to the average cross elasticity at constant change in entropy of each QC.

### 5.1. Complementarity and Substitutability of QCs

#### 5.1.1. Tertiary Catchment C11

The results of cross MAR elasticties are displayed in Table 2 and Table 3. As in economics related studies [14,36], the two matrices were found to be asymmetric as shown in these tables. The asymmetry shows the degree of complementarity or substitutability in terms of water resource utilization within the same QC pair. For example, the cross elasticity values for the pair (C11A, C11B) were 0.3 and 1. It was observed from Table 2 and Table 3 that all the entries (self-elasticities) in the upper left diagonal were positive and acceptable for the zone of functioning resilience. In the above-mentioned tables, it was revealed that the cross MAR elasticities were either complements or substitutes and were not necessarily always in the zone of functioning resilience.

For instance, between 1990 and 2005, and between 2005 and 2012, the QC pair (C11A, C11B) was in the zone of functioning resilience. Since the cross elasticities were positive, they could be considered as substitutes with different strengths, i.e., between 1990 and 2005, a decrease of 10% in MAR for catchment C11A, corresponded to a 10% decrease in entropy for C11B, whereas a decrease of 10% in MAR for C11B was associated with 3.6% decrease in entropy for C11A. It could mean that water resources for these two QCs could still be used interchangeably in a sustainable manner, at different periods. However, the level of catchment resilience for C11A was relatively slightly higher than that for C11B. Between 2005 and 2012, a decrease of 10% in MAR for C11A (C11B) implied almost the same decrease in entropy for C11B (C11A).

Between 1990 and 2005, for the pair of complements (C11F, C11K), it was observed that a decrease of 10% in MAR for catchment C11K, corresponded to a decrease of 1.2% in entropy for C11F whereas a decrease of 10% in MAR for C11F was associated with 33% decrease in entropy for C11K. Although these two QCs could be substitutes, it could mean that the change in MAR for C11K was associated with the zone of functioning resilience for C11F, while the opposite implied promptness to vulnerability for C11K. This could be an alarming situation for water managers, of the implications of a decrease in MAR for C11F. This situation would coincide with the zone of uniformity; where water resources were regarded as unsustainable [11]. Between 2005 and 2012; 10% increase in MAR for C11F was associated with 44% increase in entropy for C11K. In the same perspective, between 2005 and 2012, it was observed that a 10% increase in MAR (for QCs, from C11A to C11G), corresponded to an increase between 33 and 61% in entropy for C11K. This situation would correspond to water resources vulnerability for C11K, i.e., with C11K operating in the zone of high entropy [11]. Overall, C11K was subjected to higher increase and decrease in uncertainty, when slight changes in MAR occurred in other catchments. A small positive constant change in MAR for C11G yielded to strong positive cross elasticities of 66%, 93%, 29% and 116% for C11H, C11J, C11L and C11M respectively. It could be suggested that these QCs were in the zone of high uncertainty or low resilience. The cause of these higher cross MAR pseudo-elasticities could be further investigated in the natural factors, e.g., climatic variations (temperature, rainfall, evapotranspiration), natural vegetation, natural wetlands and aquifers, soil type, topography, etc. The effect of unquantifiable land use changes should be taken into consideration. On the other hand, the cause of relatively low cross MAR elasticities could be investigated in the combined effect among natural conditions. This combined effect could be complex with the increasing number of factors. They could influence positively or negatively cross MAR pseudo-elasticities. Previous studies on hydrological elasticity [2,9,11,29,30,31], showed such an influence. A similar analysis could be extended to the rest of substitutes presented in the tables.

The results in Table 2 and Table 3 revealed also that C11A and C11L were substitutable QCs between 2005 and 2012 and displayed an acceptable level of resilience for water resources. However, they were complementary QCs with different strengths, i.e., between 1990 and 2005, a decrease of 10% in MAR for catchment C11A, corresponded to an increase of 3.8% in entropy for C11L, whereas an increase of 10% in MAR for C11L was associated with 8.6% decrease in entropy for C11A. These results could mean that the relatively high uniform distribution of MAR for C11L (i.e., entropy increase) could be an indication of sustaining water resources. Water resources from C11L could be used as a complement of the yield from C11A to balance water demand/supply. Between 2005 and 2012, an increase of 10% in MAR for catchment C11A, corresponded to a decrease of 4.6% in entropy for C11L, whereas a decrease of 10% in MAR for C11L was associated with 6.7% increase in entropy for C11A. It could mean that a relatively high yield could be available for C11A, and hence used to assure the sustainability of water resources. Water managers could use more water from C11A than from C11L to balance water demand/supply. Water resources from C11A could be used to complement MAR for C11L.

Between 2005 and 2012, all cross MAR elasticity values were relatively weak (low) and implied that all complements could maintain a good level of resilience for water resources. This was not the case between 1990 and 2005; where there were instances of vulnerability of water resources for selected QC pairs. Hence complements displayed elastic instability, between 1990 and 2005, whereas they were elastically stable between 2005 and 2012.

#### 5.1.2. Tertiary Catchment C12

According to the results displayed in Table 4 and Table 5, there were complementary and substitutable QC pairs in C12. In addition, there were QC pairs which displayed both positive and negative cross MAR elasticities, hence having a mixture of complement and substitute in the same QC pairs. Between 1990 and 2005, all QCs had relatively low cross MAR elasticities, hence displayed an acceptable level of resilience, except for few QCs pairs between 2005 and 2012. All substitutes had relatively low cross elasticities, hence displayed an acceptable level of resilience. The implications in terms of water sustainability are similar to substitutes as discussed for TC C11.

Between 2005 and 2012, for the pair (C12A, C12D), 10% increase in MAR for C12D, led to 45% decrease in entropy for C12A, while 10% decrease in MAR for C12A, led to 1% increase in entropy for C12D. The increase in MAR for C12D might cause water resources for C12A to be elastically unstable. Hence a change in MAR from catchments other than C12D could be considered to maintain the level of acceptable resilience. 

It was observed in selected QC pairs that one catchment was a substitute while the other was complement and vice-versa. Just an illustration; e.g., between 1990 and 2005, and between 2005 and 2012, the QC pair (C12E, C12A) was in the zone of functioning resilience. Interestingly, between 1990 and 2005, both C12A and C12E were substitutes; however between 2005 and 2012, C12E was a complement of C12A and C12A was a substitute of C12E.

Between 2005 and 2012, a decrease of 10% in MAR for catchment C12A, corresponded to an increase of 0.1% in entropy for C12E, whereas a decrease of 10% in MAR for C12E was associated with 16% decrease in entropy for C12A. It is believed that an increase in entropy for C12E could help have fairly a good distribution of MAR in the pair, for water resource sustainability. Hence water resources for C12E could be used as a complement to balance water demand/supply from C12A. When MAR for C12E decreases, water resources from C12A could be used as a substitute for C12E, without compromising the zone of acceptable uncertainty. 

#### 5.1.3. Tertiary Catchment C13

From 1990 to 2012, the results in Table 6 and Table 7 revealed that the complementary QC pairs displayed very low cross elasticities, i.e., close to 0. Hence, a variation of MAR for a given QC was insignificant to cause an opposite variation in uncertainty for another QC. These weak cross elasticities could mean that the water resources from QCs could be used jointly to maintain high resilience. 

Similar to complements, very low cross MAR elasticities were observed for substitutable QC pairs. As shown in Table 6 and Table 7, variations of MAR for specific QCs caused in the same direction, insignificant variations of uncertainty for other QCs. Water resources from those specific QCs could be used as substitutes of other QCs and still maintaining a high level of resilience. The results in these tables showed that there was a mixture of complement and substitute in selected QC pairs. The implications of this mixture on water sustainability can be interpreted as done earlier.

#### 5.1.4. Tertiary Catchment C21

As displayed in Table 8 and Table 9, the values of cross MAR elasticities were relatively low; hence catchments could operate in the zone of tolerable entropy. Complementary, substitutable QC pairs as well as a mixture of complement and substitute in specific QC pairs were observed. 

#### 5.1.5. Tertiary Catchment C22

The results of cross MAR elasticities were summarized in Table 10 and Table 11. Weak cross elasticities were observed, from 2005 and 2012, however cases of high elastic instability of water resources could be observed from 1990 to 2005 due to high changes in entropy associated with a constant change in MAR for C22E. Water management could focus on constant changes in MAR for QCs other than C22E to avoid elastic instability, in terms of water resources. 

#### 5.1.6. Tertiary Catchment C23

From 1990 to 2005, low cross elasticities were observed as displayed in Table 12 and Table 13. However, from 2005 and 2012, cases of low catchment resilience could be observed due to high changes in entropy associated with a constant change in MAR for C23F; except relatively low changes in uncertainty were observed for C23J, C23K and C23L. In situations where a change in MAR for C23F remained constant, water management could focus more on C23J, C23K and C23L for water resource sustainability. Alternatively, a constant change in MAR for QCs other than C23F should be considered to assure elastic stability in catchment yields.

#### 5.1.7. Tertiary Catchment C81

As depicted in Table 14 and Table 15, several cases of very low resilience for QCs were observed between 1990 and 2005. C81M and C81C had the highest cross MAR elasticity; that is 210% change in entropy for C81M was associated with 10% change in MAR for C81C. During the same period, TC C81 scored cases of vulnerability of water resources for C81A, C81B, C81C, C81F and C81L, due to relatively high self-elasticities. QC pairs in C81 which do not fall in the range of acceptable resilience would need particular attention as far as water resources management is concerned. Between 2005 and 2012, cases of high elastic instability were observed only for a constant value in entropy for C81D associated with different changes in MAR for other QCs.

#### 5.1.8. Tertiary Catchment C82

Low cross MAR elasticity values were observed, from 1990 to 2005, and from 2005 and 2012, as shown in Table 16 and Table 17 respectively. However, between 1990 to 2005 a constant decrease in MAR for C82H yielded high values of cross elasticity (high elastic instability). Between 2005 and 2012, a decrease in MAR for C82B, was linked to a higher increase in uncertainty for C82A, C82B, C82E and C82F. Likewise, an increase in MAR for C82F was associated with a drastic decrease in uncertainty for C82G, which could yield water resource vulnerability, within the zone of uniformity. 

#### 5.1.9. Tertiary Catchment C83

Between 2005 and 2012, the results in Table 18 and Table 19, displayed weak cross elasticities. However, between 1990 and 2005, a constant decrease in MAR for C83A, yielded elastic instability, with C83B, C83C, C83E, C83F, C83J and C83M, due to high entropy in these QCs. Similarly, a change in MAR for C83J, yielded vulnerable water resources with all QCs, except C83D, C83G and C83H. It was also observed that high self-elasticity values were recorded for C83A, C83B and C83J. Hence a decrease in MAR for these QCS could be associated with unsustainable water resources in the same QCs.

### 5.2. Dominance of Complements or Substitutes

**Figure 3:**
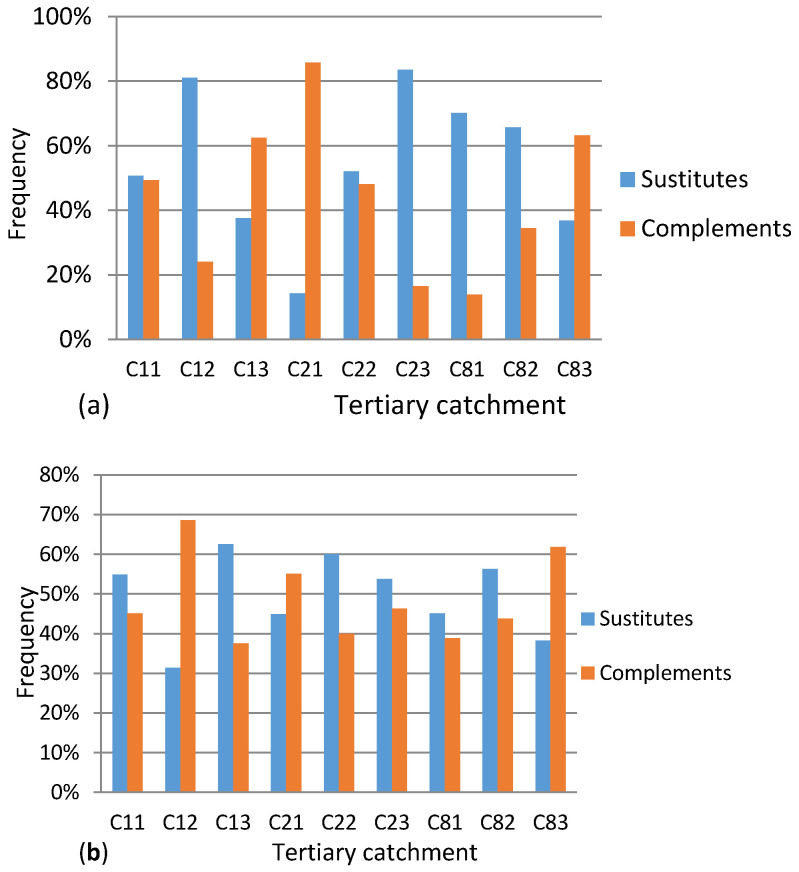
(**a**) Cross mean annual elasticity dominance between 1990 and 2005. (**b**) Cross mean annual runoff elasticity dominance between 2005 and 2012.

The number of complements and substitutes per TC were computed and their corresponding frequencies within the TC were summarized in Figure 3a,b. It could be observed that, in most TCs, substitutable QCs dominated over complementary QCs.

### 5.3. Catchment Vulnerability (Risk)

**Figure 4:**
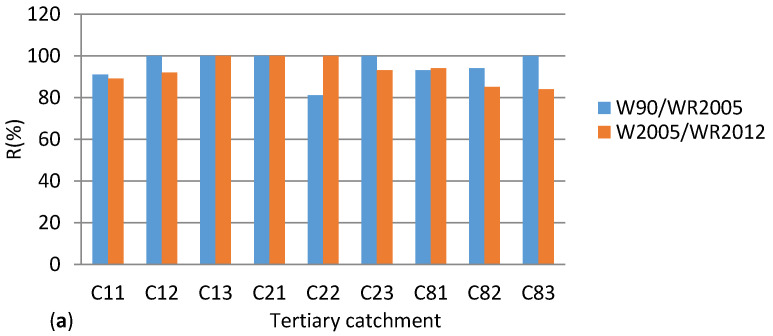

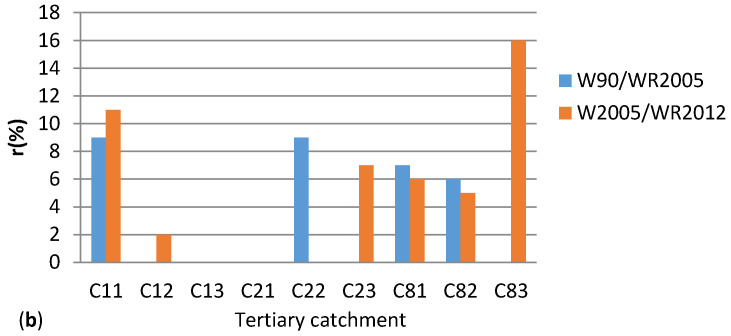
(**a**) Level of assurance of water resources sustainability (R) in tertiary catchments of Upper Vaal. (**b**) Vulnerability risk of water resources (r) in tertiary catchments of Upper Vaal.

The level of assurance for QCs to sustain water resources QCs in the Upper Vaal and the risk for not sustaining water resources were computed and presented in Figure 4a,b, respectively. It was observed that the risk associated with water resources vulnerability varied between 0 and 16%. Hence between 1990 and 2012, sustainability level (i.e., the level of assurance) to sustain water resources was relatively high and varied between 84 and 100%. Nonetheless, between 1990 and 2005, in most cases QCs displayed slightly higher level of assurance, as compared to the period between 2005 and 2012. In reality, when sustainability notion of water resources includes several variables other than MAR, it could be very likely that the values of the risk of vulnerability would increase from the above-mentioned values, hence the level of assurance to sustain water resources would decrease further.

Table 20 depicts the highest cross MAR elasticity for each TC, between 1990 and 2005 and, between 2005 and 2012. Between 1990 and 2005, the following QC pairs C11K & C11G, C22K & C22E, C81M & C81C and C83C & C83J scored their highest cross elasticity values outside the zone of functioning resilience; hence these QCs could be associated with the lowest resilience, in terms of water resources. Likewise, between 2005 and 2012, the QC pairs C11G & C11K, C12C & C11D, C23A & C23F, C81D & C81F and C82G & C82GF were associated with low resilience. For example 10% change in MAR for C11G was linked to 144% reduction and 61% increase in entropy for C11K, between 1990 and 2005 and between 2005 and 2012 respectively. On the other hand 10% change in MAR for C83J was linked to 278% increase in entropy for C83C, between 1990 and 2005. This reduction and increase in entropy implied that C11G and C11K were in the zone of elastic instability associated with a relatively smaller change in MAR as compared to the change in entropy. Situations of higher cross MAR pseudo-elasticities could be due to climatic influences on changes in MAR. Water management and planning should take care of this situation.

**Figure 5:**
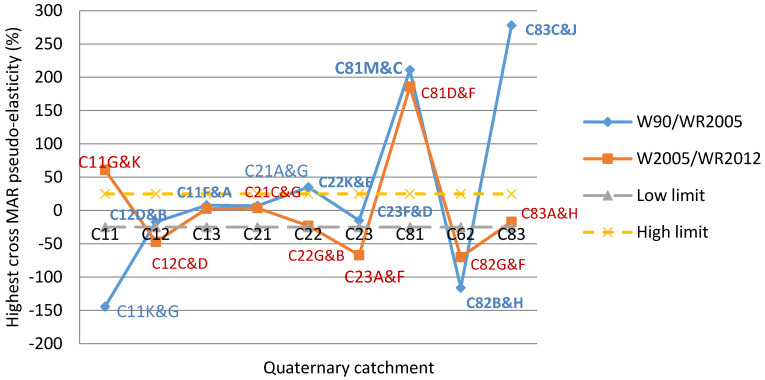
Highest cross mean annual runoff pseudo-elasticity for tertiary catchments of the Upper Vaal region.

The results in Table 20 have been plotted in Figure 5. This figure shows that selected QCs in C11, C22, C81, C82 and C83 their highest cross MAR elasticity outside the range the zone of acceptable entropy/resilience. The global highest cross MAR elasticity of entropy value was located in the tertiary catchment C83, followed by C81 and C82. These TCs could be prompt to vulnerability when their highest cross elasticity values were considered.

From the results shown in Table 20, the highest cross MAR elasticity values were derived roughly for secondary catchments C1, C2 and C3. For each secondary catchment, the QC pair was selected based on the overall highest value among three TCs. In this manner, the QC pairs were C11K & C11G, C22K & C22E and C83C & C83J respectively for C1, C2 and C3, between 1990 and 2005. Between 1990 and 2005, the QC pairs corresponding to the secondary catchments were C12C & C11D, C23A & C23F, C81D & C81F. Since these QCs scored their highest cross-elasticity values; they could be associated with a high risk of vulnerability, with regard to water resources.

### 5.4. Cross MAR Pseudo Elasticities under Constant Change in MAR/Entropy

#### 5.4.1. Constant Change in MAR

The last row of each table (i.e., from Table 2, Table 3, Table 4, Table 5, Table 6, Table 7, Table 8, Table 9, Table 10, Table 11, Table 12, Table 13, Table 14, Table 15, Table 16, Table 17, Table 18 and Table 19) showed that, on average a constant change in MAR for any QC was associated with a relatively low change in entropy for the remaining QCs, hence yielded low cross MAR elasticity values. This could lead to QCs operating in the zone of functioning resilience. However, higher values of cross MAR elasticity (low catchment resilience) were scored by a change in MAR for C81A, C81C, C81D, C81J, C83A and C83J, between 1990 and 2005. It was observed generally that on average, positive cross MAR elasticities overshadowed negative cross elasticities. For a change in MAR for a given QC, the remaining QCs could be used as substitutes of the given QC in most cases, without compromising the sustainability of water resources. Nonetheless, between 2005 and 2012, exceptions were observed for changes in MAR for C11H, C11J and C11L; hence the remaining QCs could be used as complements of these 3 QCs respectively. Between 1990 and 2005, only C11G made an exception. Similarly, between 1990 and 2005, such an exception was observed for C12D, C21B, C21C, C21D, C21E, C21F, C21G, C22A, C22B, C22C, C22D, C23E, C81G, C81H, C81J, C82B, C82F and C83A; and between 2005 and 2012 for C12D, C23D, C23E, C23F, C23G, C81C, C82A, C82D, C82E, C82F, C82G and C82H. 

#### 5.4.2. Constant Change in Entropy

The last column of each table (i.e., from Table 2, Table 3, Table 4, Table 5, Table 6, Table 7, Table 8, Table 9, Table 10, Table 11, Table 12, Table 13, Table 14, Table 15, Table 16, Table 17, Table 18 and Table 19) showed that on average, changes in MAR of QCs could lead to a constant change in entropy for any QC, within the zone of functioning resilience. However, between 2005 and 2012, cases of high cross MAR elasticities were observed for C81C and C81D; likewise, for C81F, C81L and C81M, between 1990 and 2005. On average, for a constant change in entropy for a given QC, it was observed that positive cross MAR elasticities overweighed negative cross elasticities and vice-versa. Hence, for a constant change in entropy of a given QC, the remaining QCs could be used as substitutes or complements of the given QC, without compromising the sustainability of water resources, within the TC.

### 5.5. Spatial Distributions of Cross MAR Pseudo-Elasticity of Entropy

**Figure 6:**
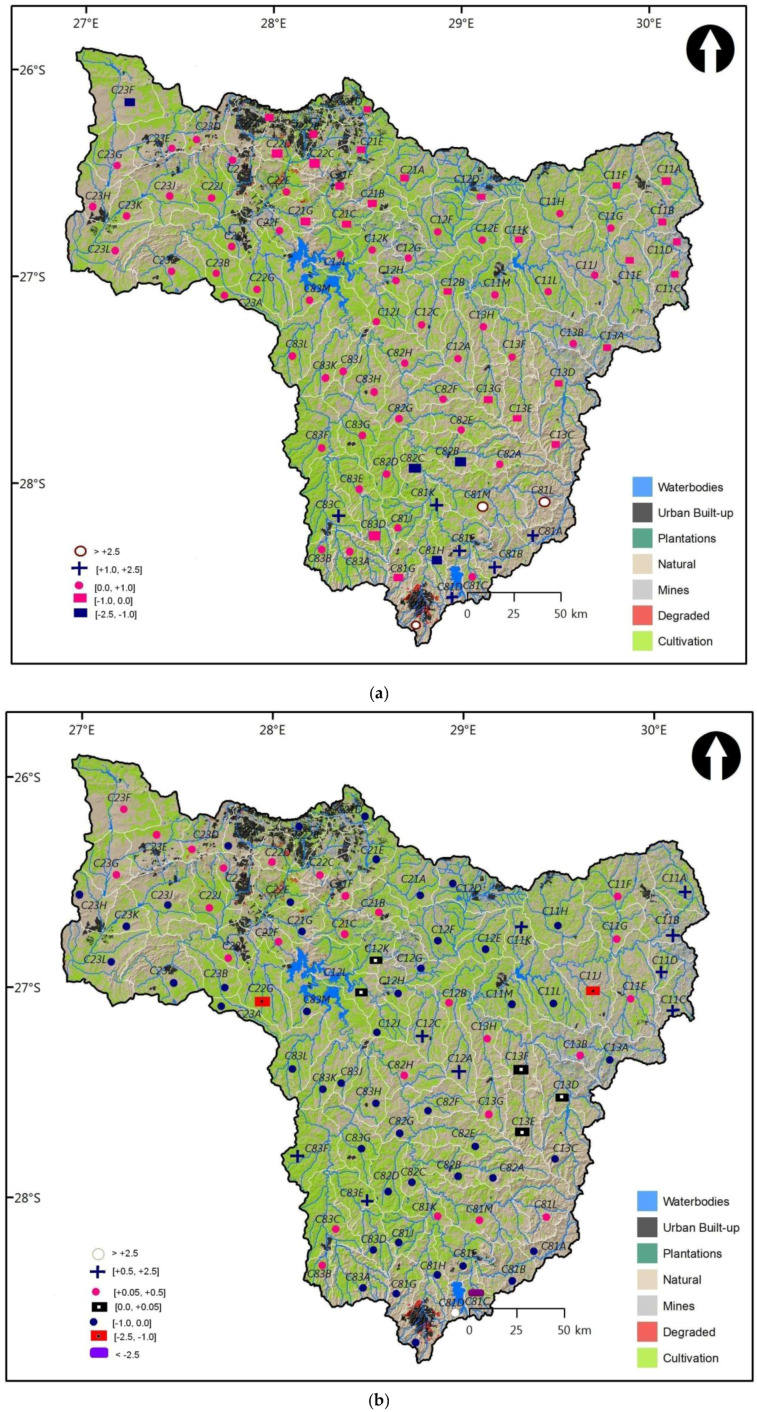
(**a**) Spatial distribution of average cross MAR pseudo-elasticities under constant entropy of QCs for WR90/WR2005. (**b**) Spatial distribution of average cross MAR pseudo-elasticities under constant entropy of QCs for WR2005/WR2012.

**Figure 7:**
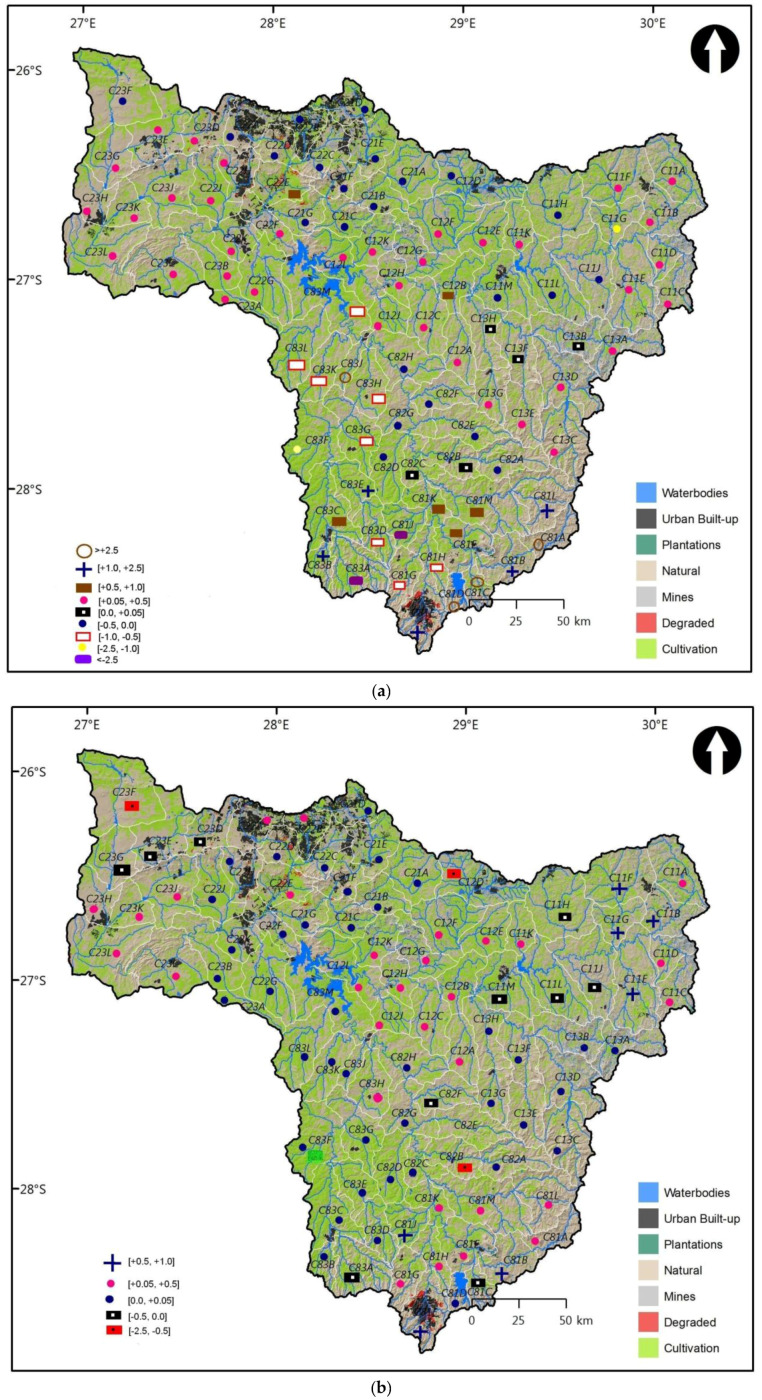
(**a**) Spatial distribution of average cross MAR pseudo-elasticities under constant MAR of QCs for WR90/WR2005. (**b**) Spatial distribution of average cross MAR pseudo-elasticities under constant MAR of QCs for WR2005/WR2012.

The spatial distribution of entropy MAR cross elasticities computed earlier in the different tables, were presented in Figure 6a,b and Figure 7a,b. For each of the following hydrological changes WR90/WR2005 and WR2005/WR2012, two cases were considered for spatial distribution, i.e., average cross MAR pseudo-elasticities under constant change in MAR and under constant entropy for a given QC. In this manner, it was easier to plot the value of cross-elasticity for each QC.

Figure 6a,b show the spatial distributions of average cross elasticities at constant entropy for the different QCs, between WR90 and WR2005; and between WR2005 and WR2012 data sets. In Figure 6a, it was observed that the majority of QCs were situated in the zone of functioning resilience since the cross elasticity values were in the interval (−2.5, 2.5). The results in this figure revealed that there were 64% of substitutable QCs as compared to 33% of complementary QCs and 3% of vulnerable QCs in the Upper Vaal. Substitutes were distributed across the majority of QCs and in all TCs. The Upper Vaal catchment could be a good location for water transfers between a given QC and its surrounding QCs of the same TC. Complements were situated mostly in the North West (urban built-up areas of the downstream of Vaal dam) and in the North East of upstream of Vaal dam. Besides water transfers, this could mean that in these locations of Upper Vaal, water could also be jointly used to balance water demand/supply in a specific QC, due to the change in MAR of the surrounding QCs, within their TC. Nonetheless, QCs associated with relatively higher cross-elasticities (>2.5) were located in the Wilge region. This region has been found to have the lowest water requirements currently and as projected in 2025 and this projection showed small differences in water requirements between 2000 and 2025 [20]. Further small increases of MAR as compared to entropy could only make more vulnerable water resources as far as a joint water use or water transfers between a specific QC and the remaining QCs are concerned. 

From Figure 6b, it was observed that 54% of complementary QCs, 44% of substitutable QCs were in the zone of acceptable resilience, while 2% QCs were in the zone of low resilience. Hence the complements dominated over substitutes, but both were distributed fairly in all TCs. This could imply that while water transfers between QCs could be promoted in the Upper Vaal, there could be a huge potential for joint water use in over 50% of QCs, to maintain a good water balance in the different TCs. Once again, it was observed that vulnerable QCs were situated in Wilge and their number was insignificant as compared to resilient QCs. However, the level of vulnerability could be very high in vulnerable QCs, as shown through the computation of cross MAR pseudo-elasticities. 

Figure 7a revealed that 57% of complementary QCs, 36% of substitutable QCs were in the zone of functionning resilience. Substitutes and complements were fairly distributed across the Upper Vaal. Although the majority of QCs were situated in the zone of functioning resilience, there were few QCs in the zone of low resilience (i.e., cross-elasticity <−2.5 or >2.5). These QCs constituted 7% and were mostly concentrated in the Wilge sub-region. For a TC of this sub-region, water resources planners should monitor relatively small changes (negative or positive) in MAR for a given QC as compared to the changes in entropy of surrounding QCs. Changes in climatic conditions (e.g., precipitation, evapotranspiration) that could cause such changes in entropy, should be assessed. A decrease in MAR and in yield could be experienced in South Africa, which is located in the South Hemisphere [41], where evaporation losses are higher than rainfall [20,42].

Figure 7b revealed that the substitutable QCs were distributed in the majority of the QCs and in all TCs, across Upper Vaal while complementary QCs were located in the North West of the downstream of dam Vaal, towards the centre of upstream of Vaal dam and the Wilge. All QCs were in the zone of acceptable resilience. Substitutes represented 86% as compared to 14% of complements. This showed a strong dominance of substitutes. Hence water transfer could prevail as compared to joint uses of water within the Upper Vaal. The spatial differences in cross MAR pseudo-elasticities for the Upper Vaal did not give any information related to natural factors such as soil, natural vegetation, topology, aquifers, etc. 

### 5.6. Overall Cross MAR Elasticity of Entropy for Each Tertiary and Each Secondary Catchment in the Upper Vaal

The average cross elasticity of each TC was found to be positive and was relatively small (<1), as shown in Table 21. This showed that the different TCs were elastically stable (i.e., relatively inelastic) as far as changes in MAR were concerned. Hence water transfers, joint use of water from different QCs, etc. could result in the zone of acceptable uncertainty. Overall, the increase in MAR for the Upper Vaal catchment was associated with an increase in entropy, such that the catchment was in the zone of functioning resilience.

As presented in Table 22, the cross MAR pseudo-elasticity for each secondary catchment was roughly determined by averaging the values of cross elasticity for its TCs. The positive cross MAR pseudo-elasticities in the secondary catchments of Upper Vaal supported that substitutable QCs remained dominant within the zone of acceptable resilience for water resources. As said previously, a complete study should be conducted for cross MAR elasticity with TC pairs within the secondary catchments.

In this study, the influence of climatic parameters on cross MAR pseudo-elasticity was not investigated, but could be assumed to be the cause of changes in the naturalized MAR data sets of the Upper Vaal catchment. Besides, the influence of other natural conditions associated with the soil type, natural vegetation, natural water storages, and geology could also be informative in the determination of cross elasticity. The combined effect of climatic conditions, together with human activities could impact positively or negatively on cross MAR pseudo-elasticity. When considering the influence of human activities, historical data could be used to assess the change in cross MAR pseudo-elasticity.

## 6. Conclusions

For the first time, cross elasticity concept has been extended to hydrology and water resources using entropy concept; i.e., cross MAR pseudo-elasticity of entropy. The level of linkage between changes in MAR and changes in entropy of another (other) catchment(s) was established in the Upper Vaal. The analysis of cross MAR pseudo-elasticity of entropy was carried out on surface water resource data sets; i.e., WR90, WR2005 and WR2012. These data were in their naturalized form when extracted from WRC database and this could assume there was little influence of the human factor. Climatic controls could be associated with these data [36] as far as cross MAR elasticity was concerned. Thresholds of a linear resilience zoning [11] were extended to the assessment of cross elasticity values. The notion of cross MAR pseudo-elasticity was shown as a fair tool to assess the complementarity and substitutability for the different QCs of the TCs in the Upper Vaal region. That is, the use of water resources for some QCs could be done jointly, while in some cases, water transfer between QCs could be an option to sustain the yield (or water resources) from selected QCs, in a way to balance demand/supply in the region. However, the Wilge sub-region of Upper Vaal displayed promptness to water resources vulnerability in fewer QCs. Cross MAR elasticity characterized the correlation strength between QC pairs (weak versus high cross elasticity) to determine the resilience of catchments. Resilient QCs (in terms of water resources) displayed relatively weak cross elasticity values as opposed to vulnerable QCs, which were characterized by high cross elasticity values. In particular, instances of cross elasticities, close to 0 were observed; showing that a variation of MAR for a given QC was insignificant to cause an opposite variation in entropy for another QC. Although the resilience was acceptable in most cases, it was revealed that in some instances some QCs displayed very strong correlations. For instance, the global highest cross MAR elasticity of entropy was located in the TCs C83, followed by C81 and could go above 100% in magnitude for specific QCs. This implied that decision-makers could pay attention to water resources planning and management, and control of water infrastructures. A spatial analysis conducted on cross MAR pseudo-elasticity revealed that, in general, there was a fair distribution of complementary and substitutable QCs in the TCs. In most cases, the latter were dominant as compared to the former. Hence, this supported that water transfers between the different QCs and beyond could prevail in the Upper Vaal as it is the current situation [20]. However, this analysis did not give more information on the cross MAR elasticity differences related to the natural characteristics of the catchments. In general, it found out that the level of assurance to sustain water resources was relatively acceptable; hence low vulnerability risk of water resources, generally less than 20%. Overall, cross MAR elasticity for QCs of the Upper Vaal region yielded the zone of tolerable entropy. 

However, it should be noted that entropy through cross MAR elasticity does not show how complementary or substitutable QCs should be managed and operated, a different time scales. The concept of cross MAR pseudo-elasticity was built on MAR pseudo-elasticity, therefore it considered only one parameter; i.e., MAR. Therefore, the inclusion of variables other than runoff, such as temperature, evaporation, precipitation should be investigated. This is a preliminary study dealing specifically with the intra-tertiary catchment assessment of cross MAR elasticity for QCs. Further work could be done on cross MAR elasticity between TCs within secondary catchments and on the assessment of its implications on the Upper Vaal catchment resilience and beyond. The impacts of climatic parameters on cross MAR pseudo-elasticity and other natural conditions associated with soil type, topography, natural vegetation, natural water storages and wetlands, could be investigated. Similar to previous studies on hydrological elasticity [2,7,8,9,11,29,30,31], the impact of these factors on cross elasticities investigated individually (bivariate relationships) could be different when these impacts are considered simultaneously in a multivariate configuration. The effect of unquantifiable land use changes should be taken into consideration. The combined effect of natural conditions, together with human activities such as land use, water use, etc. could impact positively or negatively on cross MAR pseudo-elasticity and would need to be also researched when historical data are considered. The concept should be investigated when considering runoff data of different time scales, i.e., monthly, seasonal, and annual, etc. The inclusion of parameters other than MAR in the determination of catchment vulnerability risk should be investigated. The linkage between cross MAR elasticity and the existing cross entropy concept could also be researched.

## Figures and Tables

**Table 1:** The number of QCs in each tertiary catchment (TC), and their MAR, in the Upper Vaal region, as extracted from [11] and originally published by the Water Research Commission of South Africa [40].

Tertiary Catchment (TC)	Number of QC	MAR (WR90) × 10^6^ m^3^	MAR (WR2005) × 10^6^ m^3^	MAR (WR2012) × 10^6^ m^3^
C11	12	548.2	546.28	527.34
C12	11	296.7	231.32	211.96
C13	8	291.8	322.49	343.05
C21	7	141.5	89.88	98.98
C22	10	131.5	150.02	157.51
C23	11	238.7	185.68	219.00
C81	12	450.6	515.02	529.08
C82	8	198	214.82	151.72
C83	12	283.8	236.76	252.56

**Table 2:** Cross MAR elasticities for tertiary catchment C11, between 1990 and 2005.

	C11A	C11B	C11C	C11D	C11E	C11F	C11G	C11H	C11J	C11K	C11L	C11M	*ε_i_*
C11A	0.56	0.36	0.29	0.36	0.40	0.99	−4.31	−0.30	−0.25	0.22	−0.86	−0.23	−0.23
C11B	1.01	0.64	0.51	0.64	0.71	1.77	−7.74	−0.53	−0.45	0.39	−1.55	−0.41	−0.42
C11C	1.25	0.80	0.63	0.79	0.88	2.19	−9.56	−0.66	−0.55	0.48	−1.91	−0.50	−0.51
C11D	1.09	0.69	0.55	0.69	0.76	1.90	−8.29	−0.57	−0.48	0.41	−1.66	−0.43	−0.44
C11E	0.74	0.47	0.37	0.47	0.52	1.29	−5.64	−0.39	−0.33	0.28	−1.13	−0.30	−0.30
C11F	0.31	0.19	0.15	0.19	0.21	0.53	−2.33	−0.16	−0.13	0.12	−0.47	−0.12	−0.13
C11G	−0.12	−0.08	−0.06	−0.08	−0.08	−0.21	0.93	0.06	0.05	−0.05	0.18	0.05	0.05
C11H	−0.86	−0.55	−0.43	−0.54	−0.60	−1.50	6.55	0.45	0.38	−0.33	1.31	0.34	0.35
C11J	−1.22	−0.78	−0.62	−0.77	−0.85	−2.13	9.31	0.64	0.54	−0.47	1.86	0.49	0.50
C11K	1.89	1.20	0.96	1.19	1.32	3.31	−14.44	−0.99	−0.84	0.72	−2.89	−0.76	−0.78
C11L	−0.38	−0.24	−0.19	−0.24	−0.27	−0.67	2.93	0.20	0.17	−0.15	0.59	0.15	0.16
C11M	−1.51	−0.96	−0.77	−0.96	−1.06	−2.65	11.57	0.80	0.67	−0.58	2.31	0.61	0.62
*ε_j_*	0.23	0.15	0.12	0.15	0.16	0.40	−1.75	−0.12	−0.10	0.09	−0.35	−0.09	−0.09

**Table 3:** Cross MAR elasticities for tertiary catchment C11, between 2005 and 2012.

	C11A	C11B	C11C	C11D	C11E	C11F	C11G	C11H	C11J	C11K	C11L	C11M	*ε_i_*
C11A	0.92	1.03	0.82	0.95	1.24	1.11	1.54	−0.43	−0.24	0.20	−0.67	−0.34	0.51
C11B	0.97	1.08	0.86	1.01	1.31	1.17	1.62	−0.45	−0.26	0.21	−0.71	−0.36	0.54
C11C	1.09	1.21	0.97	1.13	1.47	1.32	1.82	−0.51	−0.29	0.23	−0.80	−0.41	0.60
C11D	1.08	1.21	0.97	1.12	1.47	1.31	1.82	−0.51	−0.29	0.23	−0.80	−0.41	0.60
C11E	0.70	0.78	0.63	0.72	0.94	0.85	1.18	−0.33	−0.19	0.15	−0.52	−0.26	0.39
C11F	0.80	0.89	0.72	0.83	1.09	0.97	1.34	−0.37	−0.21	0.17	−0.59	−0.30	0.44
C11G	0.84	0.93	0.75	0.86	1.14	1.01	1.40	−0.39	−0.22	0.18	−0.61	−0.31	0.46
C11H	−0.75	−0.84	−0.67	−0.78	−1.02	−0.91	−1.25	0.34	0.20	−0.16	0.55	0.28	−0.42
C11J	−1.94	−2.16	−1.73	−2.00	−2.64	−2.34	−3.23	0.89	0.51	−0.41	1.42	0.72	−1.08
C11K	3.67	4.10	3.28	3.79	5.00	4.43	6.11	−1.69	−0.96	0.79	−2.70	−1.37	2.04
C11L	−0.46	−0.51	−0.41	−0.47	−0.62	−0.55	−0.76	0.21	0.12	−0.09	0.34	0.17	−0.25
C11M	−1.39	−1.55	−1.25	−1.44	−1.90	−1.68	−2.32	0.64	0.36	−0.28	1.04	0.52	−0.77
*ε_j_*	0.46	0.51	0.41	0.48	0.62	0.56	0.77	−0.22	−0.12	0.10	−0.34	−0.17	0.26

**Table 4:** Cross MAR elasticities for tertiary catchment C12, between 1990 and 2005.

	C12A	C12B	C12C	C12D	C12E	C12F	C12G	C12H	C12J	C12K	C12L	*ε_i_*
C12A	0.20	0.72	0.18	−0.38	0.17	0.18	0.17	0.16	0.16	0.21	0.22	0.18
C12B	−0.36	−1.28	−0.32	0.67	−0.30	−0.32	−0.30	−0.29	−0.28	−0.37	−0.39	−0.32
C12C	0.31	1.11	0.27	−0.58	0.26	0.28	0.26	0.25	0.25	0.32	0.34	0.28
C12D	−0.49	−1.73	−0.43	0.91	−0.40	−0.43	−0.41	−0.40	−0.38	−0.50	−0.53	−0.43
C12E	0.38	1.33	0.33	−0.70	0.31	0.34	0.32	0.31	0.30	0.38	0.41	0.34
C12F	0.25	0.89	0.22	−0.47	0.21	0.22	0.21	0.20	0.20	0.26	0.27	0.22
C12G	0.34	1.20	0.30	−0.63	0.28	0.30	0.28	0.28	0.27	0.35	0.37	0.30
C12H	0.44	1.55	0.38	−0.82	0.36	0.39	0.37	0.36	0.34	0.45	0.47	0.39
C12J	0.48	1.70	0.42	−0.89	0.39	0.43	0.40	0.39	0.38	0.49	0.52	0.43
C12K	0.19	0.65	0.16	−0.34	0.15	0.16	0.15	0.15	0.14	0.19	0.20	0.16
C12L	0.12	0.42	0.10	−0.22	0.10	0.11	0.10	0.10	0.09	0.12	0.13	0.11
*ε_j_*	0.17	0.60	0.15	−0.31	0.14	0.15	0.14	0.14	0.13	0.17	0.18	0.15

**Table 5:** Cross MAR elasticities for tertiary catchment C12, between 2005 and 2012.

	C12A	C12B	C12C	C12D	C12E	C12F	C12G	C12H	C12J	C12K	C12L	*ε_i_*
C12A	0.50	0.66	0.46	−4.47	1.61	1.56	1.58	1.63	1.61	1.56	1.53	0.75
C12B	0.28	0.37	0.26	−2.52	0.91	0.88	0.89	0.92	0.91	0.88	0.86	0.42
C12C	0.53	0.69	0.49	−4.72	1.69	1.65	1.67	1.71	1.69	1.65	1.61	0.79
C12D	−0.10	−0.13	−0.09	0.88	−0.31	−0.31	−0.31	−0.32	−0.31	−0.31	−0.30	−0.15
C12E	−0.01	−0.02	−0.01	0.12	−0.04	−0.04	−0.04	−0.04	−0.04	−0.04	−0.04	−0.02
C12F	−0.01	−0.01	−0.01	0.06	−0.02	−0.02	−0.02	−0.02	−0.02	−0.02	−0.02	−0.01
C12G	−0.01	−0.01	−0.01	0.10	−0.04	−0.04	−0.04	−0.04	−0.04	−0.04	−0.03	−0.02
C12H	−0.02	−0.03	−0.02	0.18	−0.06	−0.06	−0.06	−0.06	−0.06	−0.06	−0.06	−0.03
C12J	−0.02	−0.02	−0.02	0.16	−0.06	−0.06	−0.06	−0.06	−0.06	−0.06	−0.05	−0.03
C12K	0.00	0.00	0.00	0.03	−0.01	−0.01	−0.01	−0.01	−0.01	−0.01	−0.01	0.00
C12L	0.00	0.00	0.00	0.02	−0.01	−0.01	−0.01	−0.01	−0.01	−0.01	−0.01	0.00
*ε_j_*	0.10	0.14	0.10	−0.92	0.33	0.32	0.33	0.34	0.33	0.32	0.32	0.15

**Table 6:** Cross MAR elasticities for tertiary catchment C13, between 1990 and 2005.

	C13A	C13B	C13C	C13D	C13E	C13F	C13G	C13H	*ε_i_*
C13A	−0.27	−0.15	−0.20	−0.16	−0.17	−0.08	−0.17	−0.13	−0.17
C13B	0.04	0.02	0.03	0.02	0.02	0.01	0.02	0.02	0.02
C13C	−0.12	−0.07	−0.09	−0.07	−0.07	−0.04	−0.07	−0.06	−0.07
C13D	−0.02	−0.01	−0.02	−0.01	−0.01	−0.01	−0.01	−0.01	−0.01
C13E	−0.03	−0.02	−0.02	−0.02	−0.02	−0.01	−0.02	−0.01	−0.02
C13F	0.81	0.46	0.59	0.50	0.50	0.24	0.51	0.38	0.50
C13G	−0.06	−0.03	−0.04	−0.03	−0.03	−0.02	−0.04	−0.03	−0.03
C13H	0.25	0.14	0.19	0.16	0.16	0.08	0.16	0.12	0.16
*ε_j_*	0.08	0.04	0.06	0.05	0.05	0.02	0.05	0.04	0.05

**Table 7:** Cross MAR elasticities for tertiary catchment C13, between 2005 and 2012.

	C13A	C13B	C13C	C13D	C13E	C13F	C13G	C13H	*ε_i_*
C13A	−0.10	−0.07	−0.09	−0.08	−0.08	−0.07	−0.07	−0.06	−0.08
C13B	0.07	0.05	0.06	0.05	0.06	0.05	0.05	0.04	0.05
C13C	−0.06	−0.04	−0.05	−0.05	−0.05	−0.04	−0.04	−0.03	−0.05
C13D	0.02	0.01	0.02	0.01	0.01	0.01	0.01	0.01	0.01
C13E	−0.01	0.00	−0.01	0.00	−0.01	0.00	0.00	0.00	0.00
C13F	0.03	0.02	0.03	0.02	0.03	0.02	0.02	0.02	0.02
C13G	0.10	0.07	0.09	0.08	0.08	0.08	0.07	0.06	0.08
C13H	0.27	0.19	0.24	0.21	0.22	0.20	0.19	0.16	0.21
*ε_j_*	0.04	0.03	0.04	0.03	0.03	0.03	0.03	0.02	0.03

**Table 8:** Cross MAR elasticities for tertiary catchment C21, between 1990 and 2005.

	C21A	C21B	C21C	C21D	C21E	C21F	C21G	*ε_i_*
C21A	0.22	0.49	0.47	0.58	0.57	0.56	0.71	0.51
C21B	−0.11	−0.23	−0.22	−0.28	−0.27	−0.26	−0.33	−0.24
C21C	−0.10	−0.22	−0.21	−0.26	−0.25	−0.25	−0.31	−0.23
C21D	−0.16	−0.35	−0.34	−0.42	−0.41	−0.40	−0.51	−0.37
C21E	−0.13	−0.28	−0.28	−0.34	−0.33	−0.32	−0.41	−0.30
C21F	−0.15	−0.32	−0.31	−0.39	−0.38	−0.37	−0.47	−0.34
C21G	−0.22	−0.49	−0.47	−0.58	−0.57	−0.56	−0.70	−0.51
*ε_j_*	−0.09	−0.20	−0.19	−0.24	−0.23	−0.23	−0.29	−0.21

**Table 9:** Cross MAR elasticities for tertiary catchment C21, between 2005 and 2012.

	C21A	C21B	C21C	C21D	C21E	C21F	C21G	*ε_i_*
C21A	−0.06	−0.04	−0.04	−0.05	−0.05	−0.04	−0.12	−0.06
C21B	0.16	0.10	0.10	0.14	0.14	0.11	0.31	0.15
C21C	0.18	0.12	0.12	0.16	0.16	0.13	0.36	0.17
C21D	−0.01	0.00	0.00	0.00	0.00	0.00	−0.01	−0.01
C21E	−0.02	−0.01	−0.01	−0.02	−0.02	−0.02	−0.04	−0.02
C21F	0.13	0.08	0.08	0.11	0.11	0.09	0.25	0.12
C21G	−0.32	−0.21	−0.21	−0.28	−0.29	−0.22	−0.64	−0.31
*ε_j_*	0.01	0.01	0.01	0.01	0.01	0.01	0.02	0.01

**Table 10:** Cross MAR elasticities for tertiary catchment C22, between 1990 and 2005.

	C22A	C22B	C22C	C22D	C22E	C22F	C22G	C22H	C22J	C22K	*ε_i_*
C22A	0.28	0.31	0.31	0.29	−2.84	−1.00	−1.06	−1.00	−0.99	−0.83	−0.65
C22B	0.30	0.33	0.33	0.31	−3.02	−1.06	−1.13	−1.07	−1.05	−0.88	−0.70
C22C	0.27	0.30	0.30	0.28	−2.77	−0.97	−1.04	−0.98	−0.97	−0.81	−0.64
C22D	0.33	0.36	0.37	0.34	−3.39	−1.19	−1.27	−1.20	−1.18	−0.99	−0.78
C22E	−0.18	−0.20	−0.20	−0.19	1.83	0.64	0.69	0.65	0.64	0.54	0.42
C22F	−0.31	−0.34	−0.34	−0.32	3.14	1.10	1.18	1.11	1.09	0.92	0.72
C22G	−0.27	−0.29	−0.30	−0.27	2.69	0.94	1.01	0.95	0.94	0.79	0.62
C22H	−0.31	−0.34	−0.35	−0.32	3.17	1.11	1.19	1.12	1.11	0.93	0.73
C22J	−0.29	−0.32	−0.33	−0.30	2.96	1.04	1.11	1.04	1.03	0.87	0.68
C22K	−0.35	−0.38	−0.39	−0.36	3.54	1.24	1.33	1.25	1.23	1.03	0.82
*ε_j_*	−0.05	−0.06	−0.06	−0.05	0.53	0.19	0.20	0.19	0.19	0.16	0.12

**Table 11:** Cross MAR elasticities for tertiary catchment C22, between 2005 and 2012.

	C22A	C22B	C22C	C22D	C22E	C22F	C22G	C22H	C22J	C22K	*ε_i_*
C22A	−0.05	−0.05	−0.03	−0.03	−0.06	−0.02	−0.02	−0.02	−0.02	−0.02	−0.03
C22B	−0.09	−0.10	−0.05	−0.06	−0.10	−0.04	−0.04	−0.04	−0.04	−0.04	−0.06
C22C	0.33	0.34	0.18	0.21	0.37	0.15	0.12	0.15	0.15	0.15	0.21
C22D	0.21	0.22	0.11	0.13	0.23	0.09	0.08	0.09	0.09	0.09	0.14
C22E	−0.14	−0.14	−0.07	−0.09	−0.15	−0.06	−0.05	−0.06	−0.06	−0.06	−0.09
C22F	0.64	0.67	0.34	0.41	0.72	0.29	0.24	0.29	0.28	0.29	0.42
C22G	−2.18	−2.30	−1.18	−1.41	−2.46	−1.00	−0.84	−0.98	−0.97	−0.98	−1.43
C22H	0.67	0.70	0.36	0.43	0.76	0.31	0.26	0.30	0.30	0.30	0.44
C22J	0.64	0.67	0.34	0.41	0.72	0.29	0.24	0.29	0.28	0.29	0.42
C22K	0.67	0.70	0.36	0.43	0.75	0.31	0.26	0.30	0.30	0.30	0.44
*ε_j_*	0.07	0.07	0.04	0.04	0.08	0.03	0.03	0.03	0.03	0.03	0.04

**Table 12:** Cross MAR elasticities for tertiary catchment C23, between 1990 and 2005.

	C23A	C23B	C23C	C23D	C23E	C23F	C23G	C23H	C23J	C23K	C23L	*ε_i_*
C23A	0.58	0.58	0.61	0.93	0.93	−0.27	0.89	0.78	0.80	0.80	0.81	0.68
C23B	0.49	0.50	0.52	0.79	0.79	−0.23	0.76	0.66	0.68	0.68	0.69	0.58
C23C	0.39	0.40	0.41	0.63	0.63	−0.18	0.60	0.53	0.54	0.54	0.55	0.46
C23D	0.12	0.13	0.13	0.20	0.20	−0.06	0.19	0.17	0.17	0.17	0.17	0.14
C23E	0.12	0.12	0.13	0.19	0.19	−0.06	0.18	0.16	0.17	0.17	0.17	0.14
C23F	−0.96	−0.97	−1.02	−1.54	−1.54	0.45	−1.48	−1.30	−1.33	−1.33	−1.35	−1.12
C23G	0.15	0.16	0.16	0.25	0.25	−0.07	0.24	0.21	0.21	0.21	0.22	0.18
C23H	0.26	0.26	0.27	0.42	0.42	−0.12	0.40	0.35	0.36	0.36	0.36	0.30
C23J	0.18	0.18	0.19	0.29	0.29	−0.09	0.28	0.25	0.25	0.25	0.26	0.21
C23K	0.23	0.23	0.24	0.37	0.37	−0.11	0.35	0.31	0.32	0.32	0.32	0.27
C23L	0.15	0.15	0.16	0.24	0.24	−0.07	0.23	0.20	0.21	0.21	0.21	0.18
*ε_j_*	0.16	0.16	0.17	0.25	0.25	−0.07	0.24	0.21	0.22	0.22	0.22	0.18

**Table 13:** Cross MAR elasticities for tertiary catchment C23, between 2005 and 2012.

	C23A	C23B	C23C	C23D	C23E	C23F	C23G	C23H	C23J	C23K	C23L	*ε_i_*
C23A	0.40	0.40	0.49	−1.73	−1.86	−6.73	−1.73	0.72	1.27	1.98	2.03	−0.51
C23B	0.31	0.32	0.38	−1.36	−1.46	−5.29	−1.36	0.57	1.00	1.56	1.60	−0.41
C23C	0.21	0.21	0.26	−0.91	−0.99	−3.56	−0.91	0.38	0.67	1.05	1.07	−0.27
C23D	−0.24	−0.24	−0.29	1.04	1.12	4.06	1.04	−0.44	−0.76	−1.20	−1.23	0.31
C23E	−0.23	−0.23	−0.28	0.98	1.06	3.83	0.98	−0.41	−0.72	−1.13	−1.15	0.29
C23F	−0.05	−0.05	−0.06	0.20	0.22	0.79	0.20	−0.08	−0.15	−0.23	−0.24	0.06
C23G	−0.24	−0.24	−0.30	1.05	1.13	4.10	1.05	−0.44	−0.77	−1.21	−1.24	0.31
C23H	0.18	0.18	0.22	−0.77	−0.83	−3.00	−0.77	0.32	0.57	0.88	0.91	−0.23
C23J	0.05	0.05	0.06	−0.22	−0.24	−0.86	−0.22	0.09	0.16	0.25	0.26	−0.07
C23K	0.01	0.01	0.01	−0.05	−0.05	−0.18	−0.05	0.02	0.03	0.05	0.05	−0.01
C23L	0.01	0.01	0.01	−0.03	−0.03	−0.10	−0.03	0.01	0.02	0.03	0.03	−0.01
*ε_j_*	0.04	0.04	0.05	−0.16	−0.17	−0.63	−0.16	0.07	0.12	0.19	0.19	−0.05

**Table 14:** Cross MAR elasticities for tertiary catchment C81, between 1990 and 2005.

	C81A	C81B	C81C	C81D	C81E	C81F	C81G	C81H	C81J	C81K	C81L	C81M	*ε_i_*
C81A	4.06	2.51	16.42	5.02	1.38	2.02	−1.35	−0.99	−5.98	0.95	2.26	1.29	2.30
C81B	4.20	2.59	16.97	5.19	1.42	2.09	−1.40	−1.03	−6.18	0.98	2.34	1.34	2.38
C81C	1.62	1.00	6.52	2.00	0.55	0.80	−0.54	−0.39	−2.38	0.38	0.90	0.51	0.91
C81D	1.86	1.14	7.50	2.29	0.63	0.92	−0.62	−0.45	−2.73	0.43	1.03	0.59	1.05
C81E	4.12	2.54	16.64	5.09	1.39	2.05	−1.37	−1.01	−6.06	0.96	2.29	1.31	2.33
C81F	6.64	4.09	26.80	8.19	2.25	3.30	−2.20	−1.62	−9.76	1.55	3.69	2.11	3.75
C81G	−0.92	−0.57	−3.72	−1.14	−0.31	−0.46	0.31	0.22	1.35	−0.21	−0.51	−0.29	−0.52
C81H	−2.42	−1.49	−9.77	−2.99	−0.82	−1.20	0.80	0.59	3.56	−0.56	−1.35	−0.77	−1.37
C81J	0.49	0.30	1.98	0.61	0.17	0.24	−0.16	−0.12	−0.72	0.11	0.27	0.16	0.28
C81K	4.23	2.61	17.10	5.23	1.43	2.11	−1.41	−1.03	−6.23	0.99	2.36	1.35	2.39
C81L	4.93	3.04	19.91	6.09	1.67	2.45	−1.64	−1.20	−7.25	1.15	2.74	1.57	2.79
C81M	5.22	3.22	21.08	6.44	1.77	2.60	−1.73	−1.27	−7.68	1.22	2.91	1.66	2.95
*ε_j_*	2.84	1.75	11.45	3.50	0.96	1.41	−0.94	−0.69	−4.17	0.66	1.58	0.90	1.60

**Table 15:** Cross MAR elasticities for tertiary catchment C81, between 2005 and 2012.

	C81A	C81B	C81C	C81D	C81E	C81F	C81G	C81H	C81J	C81K	C81L	C81M	*ε_i_*
C81A	−0.30	−0.40	0.06	−0.01	−0.29	−0.46	−0.22	−0.21	−0.41	−0.13	−0.17	−0.16	−0.23
C81B	−0.45	−0.62	0.09	−0.02	−0.44	−0.70	−0.34	−0.32	−0.63	−0.20	−0.26	−0.24	−0.34
C81C	−4.68	−6.37	0.90	−0.23	−4.52	−7.24	−3.52	−3.29	−6.53	−2.05	−2.68	−2.47	−3.56
C81D	12.05	16.39	−2.33	0.59	11.64	18.63	9.07	8.47	16.80	5.27	6.89	6.36	9.15
C81E	−0.34	−0.46	0.07	−0.02	−0.33	−0.52	−0.25	−0.24	−0.47	−0.15	−0.19	−0.18	−0.26
C81F	−0.34	−0.47	0.07	−0.02	−0.33	−0.53	−0.26	−0.24	−0.48	−0.15	−0.20	−0.18	−0.26
C81G	−0.19	−0.26	0.04	−0.01	−0.18	−0.30	−0.14	−0.13	−0.27	−0.08	−0.11	−0.10	−0.15
C81H	−0.13	−0.18	0.03	−0.01	−0.13	−0.20	−0.10	−0.09	−0.18	−0.06	−0.08	−0.07	−0.10
C81J	−0.63	−0.86	0.12	−0.03	−0.61	−0.98	−0.48	−0.45	−0.88	−0.28	−0.36	−0.33	−0.48
C81K	0.46	0.63	−0.09	0.02	0.45	0.72	0.35	0.33	0.65	0.20	0.27	0.25	0.35
C81L	0.07	0.09	−0.01	0.00	0.06	0.10	0.05	0.05	0.09	0.03	0.04	0.04	0.05
C81M	0.14	0.19	−0.03	0.01	0.13	0.21	0.10	0.10	0.19	0.06	0.08	0.07	0.11
*ε_j_*	0.47	0.64	−0.09	0.02	0.45	0.73	0.35	0.33	0.66	0.21	0.27	0.25	0.36

**Table 16:** Cross MAR elasticities for tertiary catchment C82 between 1990 and 2005.

	C82A	C82B	C82C	C82D	C82E	C82F	C82G	C82H	*ε_i_*
C82A	0.42	−0.14	−0.22	0.32	0.05	0.51	0.60	3.33	0.61
C82B	−1.46	0.50	0.75	−1.11	−0.17	−1.77	−2.09	−11.59	−2.12
C82C	−1.19	0.40	0.62	−0.91	−0.14	−1.45	−1.70	−9.45	−1.73
C82D	0.68	−0.23	−0.35	0.52	0.08	0.83	0.98	5.42	0.99
C82E	0.34	−0.12	−0.18	0.26	0.04	0.42	0.49	2.74	0.50
C82F	0.41	−0.14	−0.21	0.31	0.05	0.50	0.59	3.28	0.60
C82G	0.30	−0.10	−0.16	0.23	0.04	0.37	0.43	2.40	0.44
C82H	0.01	0.00	0.00	0.01	0.00	0.01	0.01	0.07	0.01
*ε_j_*	−0.06	0.02	0.03	−0.05	−0.01	−0.07	−0.09	−0.47	−0.09

**Table 17:** Cross MAR elasticities for tertiary catchment C82 between 2005 and 2012.

	C82A	C82B	C82C	C82D	C82E	C82F	C82G	C82H	*ε_i_*
C82A	−0.17	−2.72	−0.10	−0.12	−0.12	1.24	−0.07	−0.11	−0.27
C82B	−0.24	−2.81	−0.14	−0.17	−0.18	1.81	−0.10	−0.17	−0.25
C82C	0.32	−1.08	0.19	0.22	0.24	−2.42	0.13	0.22	−0.27
C82D	0.11	−1.24	0.07	0.08	0.09	−0.86	0.05	0.08	−0.20
C82E	0.04	−2.76	0.02	0.03	0.03	−0.29	0.02	0.03	−0.36
C82F	−0.93	−4.44	−0.55	−0.65	−0.70	6.99	−0.37	−0.64	−0.16
C82G	0.93	0.62	0.55	0.64	0.69	−6.92	0.37	0.64	−0.31
C82H	0.11	1.62	0.07	0.08	0.08	−0.84	0.04	0.08	0.16
*ε_j_*	0.02	−1.60	0.01	0.01	0.02	−0.16	0.01	0.01	−0.21

**Table 18:** Cross MAR elasticities for tertiary catchment C83 between 1990 and 2005.

	C83A	C83B	C83C	C83D	C83E	C83F	C83G	C83H	C83J	C83K	C83L	C83M	*ε_i_*
C83A	−5.53	1.84	0.98	−0.88	1.62	−1.44	−0.83	−0.83	16.04	−1.02	−1.02	−1.00	0.66
C83B	−7.84	2.60	1.40	−1.24	2.30	−2.05	−1.18	−1.17	22.75	−1.45	−1.44	−1.42	0.94
C83C	−9.58	3.18	1.71	−1.52	2.81	−2.51	−1.44	−1.44	27.82	−1.77	−1.76	−1.74	1.15
C83D	0.26	−0.09	−0.05	0.04	−0.08	0.0	0.04	0.04	−0.75	0.05	0.05	0.05	−0.03
C83E	−8.19	2.72	1.46	−1.30	2.40	−2.14	−1.23	−1.23	23.77	−1.51	−1.51	−1.49	0.98
C83F	−3.30	1.10	0.59	−0.52	0.97	−0.86	−0.50	−0.49	9.57	−0.61	−0.61	−0.60	0.39
C83G	−0.50	0.17	0.09	−0.08	0.15	−0.13	−0.08	−0.07	1.45	−0.09	−0.09	−0.09	0.06
C83H	−0.07	0.02	0.01	−0.01	0.02	−0.02	−0.01	−0.01	0.20	−0.01	−0.01	−0.01	0.01
C83J	−5.53	1.84	0.99	−0.88	1.62	−1.45	−0.83	−0.83	16.06	−1.02	−1.02	−1.00	0.66
C83K	−1.52	0.51	0.27	−0.24	0.45	−0.40	−0.23	−0.23	4.42	−0.28	−0.28	−0.28	0.18
C83L	−2.49	0.83	0.44	−0.39	0.73	−0.65	−0.37	−0.37	7.23	−0.46	−0.46	−0.45	0.30
C83M	−3.21	1.07	0.57	−0.51	0.94	−0.84	−0.48	−0.48	9.33	−0.59	−0.59	−0.58	0.38
*ε_j_*	−3.96	1.32	0.71	−0.63	1.16	−1.03	−0.59	−0.59	11.49	−0.73	−0.73	−0.72	0.47

**Table 19:** Cross MAR elasticities for tertiary catchment C83 between 2005 and 2012.

	C83A	C83B	C83C	C83D	C83E	C83F	C83G	C83H	C83J	C83K	C83L	C83M	*ε_i_*
C83A	0.97	−0.74	−0.60	−1.15	−0.51	−0.43	−1.23	−1.65	−1.37	−1.30	−1.44	−1.32	−0.90
C83B	−0.28	0.22	0.18	0.34	0.15	0.13	0.36	0.48	0.40	0.38	0.42	0.39	0.26
C83C	−0.34	0.26	0.21	0.40	0.18	0.15	0.43	0.58	0.48	0.46	0.50	0.46	0.31
C83D	0.02	−0.01	−0.01	−0.02	−0.01	−0.01	−0.02	−0.03	−0.03	−0.03	−0.03	−0.03	−0.02
C83E	−0.60	0.46	0.37	0.71	0.32	0.26	0.76	1.02	0.84	0.80	0.89	0.81	0.55
C83F	−0.70	0.53	0.43	0.83	0.37	0.31	0.89	1.19	0.99	0.94	1.04	0.95	0.65
C83G	0.05	−0.04	−0.03	−0.06	−0.03	−0.02	−0.07	−0.09	−0.07	−0.07	−0.08	−0.07	−0.05
C83H	0.18	−0.13	−0.11	−0.21	−0.09	−0.08	−0.22	−0.30	−0.25	−0.24	−0.26	−0.24	−0.16
C83J	0.11	−0.08	−0.07	−0.13	−0.06	−0.05	−0.14	−0.19	−0.16	−0.15	−0.16	−0.15	−0.10
C83K	0.08	−0.06	−0.05	−0.09	−0.04	−0.03	−0.10	−0.13	−0.11	−0.10	−0.11	−0.10	−0.07
C83L	0.10	−0.08	−0.06	−0.12	−0.05	−0.05	−0.13	−0.18	−0.14	−0.14	−0.15	−0.14	−0.10
C83M	0.07	−0.05	−0.04	−0.08	−0.03	−0.03	−0.08	−0.11	−0.09	−0.09	−0.10	−0.09	−0.06
*ε_j_*	−0.03	0.02	0.02	0.03	0.02	0.01	0.04	0.05	0.04	0.04	0.04	0.04	0.03

**Table 20:** Highest cross mean annual runoff pseudo-elasticity of QCs in TCs of the Upper Vaal region.

Tertiary Catchment	Highest Cross MAR Elasticity (%)	
	W90/WR2005 (Quaternary Catchment Pair)	W2005/WR2012 (Quaternary Catchment Pair)
C11	−144.4 (C11K & C11G)	61 (C11G & C11K)
C12	−17.2 (C12D & C12B)	−47.1 (C12C & C11D)
C13	8 (C13F & C13A)	2.7 (C13F & C11A)
C21	7.1 (C21A & C11G)	3.6 (C21C & C11G)
C22	35 (C22K & C22E)	−23 (C22G & C22B)
C23	−15 (C23F & C23D)	−67 (C23A & C23F)
C81	211 (C81M & C81C)	186 (C81D & C81F)
C82	−116 (C82B & C82H)	−70 (C82G & C82GF)
C83	278(C83C & C83J)	−17(C83A & C83H)

**Table 21:** Overall average cross MAR pseudo elasticity for tertiary catchments of Upper Vaal.

Tertiary Catchment	Elasticity [-]	
	WR90/2005	WR2005/WR2012
C11	−0.094	0.26
C12	0.15	0.16
C13	0.046	0.032
C21	−0.211	0.08
C22	0.122	0.045
C23	0.183	−0.048
C81	1.604	0.36
C82	−0.087	−0.209
C83	0.47	0.027

**Table 22:** Cross MAR pseudo elasticity for secondary catchments of Upper Vaal.

Secondary Catchment	Elasticity [-]	
	WR90/2005	WR2005/WR2012
Upstream of Vaal dam (C1)	0.03	0.15
Downstream of Vaal dam (C2)	0.03	0.077
Wilge (C8)	0.66	0.059

## References

[1] Sankarasubramanian A., Vogel R.M., Limbrunner J.F. (2001). Climate elasticity of streamflow in the United States. Water Resour. Res..

[2] Chiew F.H.S. (2006). Estimation of rainfall elasticity of streamflow in Australia. Hydrol. Sci. J..

[3] Andréassian V., Coron L., Lerat J., Le Moine N. (2016). Climate elasticity of streamflow revisited—An elasticity index based on long-term hydrometeorological records. Hydrol. Earth Syst. Sci..

[4] Mulugeta D., Greenfield J., Bolen T., Conley L., Health C. Price- and cross-price elasticity estimation using SAS. Proceedings of the SAS Global Forum 2013.

[5] Santeramo F.G. (2017). Cross-Price Elasticities for Oils and Fats in the US and the EU. http://www.theicct.org/sites/default/files/publications/Cross-price-elasticities-for-oils-fats-US-EU_ICCT_consultant-report_06032017.pdf.

[6] Nowak W.P., Savage I. (2013). The cross elasticity between gasoline prices and transit use: Evidence from Chicago. Transp. Policy.

[7] Fu G., Charles S.P., Chiew F.H.S. (2007). A two-parameter climate elasticity of streamflow index to assess climate change effects on annual streamflow. Water Resour. Res..

[8] Vano J.A., Das T., Lettenmaier D.P. (2012). Hydrologic sensitivities of Colorado River runoff to changes in precipitation and Temperature. J. Hydrometeorol..

[9] Meng F., Su F., Yang D., Tong K., Hao Z. (2016). Impacts of recent climate change on the hydrology in the source region of the Yellow River basin. J. Hydrol. Reg. Stud..

[10] Allaire M.C., Vogel R.M., Kroll C.N. (2015). The hydro-morphology of an urbanizing watershed using multivariate elasticity. Adv. Water Resour..

[11] Ilunga M. (2017). Assessing catchment resilience using entropy associated with mean annual runoff for the Upper Vaal catchment in South Africa. Entropy.

[12] Taragin C., Sandfort M. (2015). The Antitrust Package. https://cran.r-project.org/web/packages/antitrust/vignettes/manual.pdf.

[13] Buklin R.E., Russel G.J., Srinivasan V. (1998). A relationship between market share elasticities and brand switching probabilities. J. Mark. Res..

[14] Song I., Chintagunta P.K. (2006). Measuring cross-category price effects with aggregate store data. Manag. Sci..

[15] Cooper L.G. (1988). Competitive maps: The structure underlying asymmetric cross elasticities. Manag. Sci..

[16] Kushkuley A., Wu S.-M. (2014). A note on Lerner Index, cross-elasticity and revenue optimization Invariants. arXiv.

[17] Russel G.J. (1992). A model of latent symmetry in cross price elasticities. Mark. Lett..

[18] Sanders S., Weisman D.L., Li D. (2008). Child safety seats on commercial airliners: A demonstration of cross-price elasticities. J. Econ. Educ..

[19] Tambe V.J., Joshi S.K. (2015). Estimating price elasticity of electricity for the major consumer categories of Gujarat State. J. Electr. Eng..

[20] Department of Water Affairs (2003). Upper Vaal Management Area: Overview of Water Resources Availability and Utilisation.

[21] National Water Resource Strategy (2013). Department of Water Affairs (Pretoria: DWA), South Africa. http://www.dwa.gov.za/nwrs/NWRS2013.aspx.

[22] Wrzesinski D. (2016). Use of Entropy in the Assessment of uncertainty of river runoff Regime in Poland. Acta Geophys..

[23] Wrzesinski D. (2013). Uncertainty of flow regime characteristics of rivers in Europe. Quaest. Geogr..

[24] Zhou F., Xu Y.P., Chen Y., Xu C.Y., Gao Y.Q., Du J.K. (2013). Hydrological response to urbanisation at different spatio-temporal scales simulated by coupling of CLUE-S and the SWAT model in the Yangtze River Delta region. J. Hydrol..

[25] Fan J., Huang Q., Chang J., Sun D., Cui S. (2013). Detecting Abrupt change of streamflow at Lintong Station of Wei River. Math. Probl. Eng..

[26] Pan S., Liu D., Wang Z., Zhao Q., Zou H., Hou Y., Liu P., Xiong L. (2017). Runoff responses to climate and land use/cover changes under future scenarios. Water.

[27] Ilunga M., Singh V.P. (2015). Measuring spatial variability of land use associated with hydrological impact in urbanized quaternary catchment using entropy. Water SA.

[28] Zhang L., Singh V.P. (2012). Bivariate rainfall and runoff analysis using entropy and Copula theories. Entropy.

[29] Chiew F.H.S., Peel M.C., McMahon T.A., Siriwardena L.W. (2006). Precipitation elasticity of streamflow in catchments across the world. Proceedings of the Fifth FRIEND World Conference.

[30] Zhou X., Zhang Y., Yang Y. (2015). Comparison of two approaches for estimating precipitation elasticity of Streamflow in China’s Main River Basins. Adv. Meteorol..

[31] Konapala G., Ashok K., Mishra A.K. (2016). Three-parameter-based streamflow elasticity model: Application to MOPEX basins in the USA at annual and seasonal scales. Hydrol. Earth Syst. Sci..

[32] Middleton B.J., Bailey A.K. (2011). Water Resources of South Africa.

[33] Midgley D.C., Pitman W.V., Middleton B.J. (1994). Surface Water Resources of South Africa 1990.

[34] Atieh M., Rudra R., Gharabaghi B., Lubitz D. (2017). Investigating the spatial and temporal variability of Precipitation using Entropy Theory. J. Water Manag. Model..

[35] Wei C., Yeh H.-C., Chen Y.-C. (2014). Spatiotemporal scaling effect on rainfall network design using entropy. Entropy.

[36] Liu Q., Yang Z., Cui B. (2008). Spatial and temporal variability of annual precipitation during 1961–2006 in Yellow River Basin, China. J. Hydrol..

[37] Mishra A.K., Mehmet O., Singh V.P. (2009). An entropy based investigation into the variability of precipitation. J. Hydrol..

[38] Price D.W., Mittelhammer R.C. (1979). A matrix of demand elasticities for fresh fruit. West. J. Agric. Econ..

[39] Houck J.P. (1966). A Look at flexibilities and elasticities. J. Farm Econ..

[40] Water Resources of South Africa 2012 Study (WR2012). http://waterresourceswr2012.co.za/.

[41] McMahon T.A., PeeL M.C., Pegram G.G.S., Smith I.N. (2011). A Simple Methodology for Estimating Mean and Variability of Annual Runoff and Reservoir Yield under Present and Future Climates. J. Hydrometeorol..

[42] Jury M.R. (2001). Economic Impacts of Climate Variability in South Africa and Development of Resource Prediction Models. J. Meteorol..

